# Methane production via photocatalytic degradation of glucose on PtO_x_ and PdO_x_-loaded TiO_2_

**DOI:** 10.1038/s41598-025-30321-w

**Published:** 2025-12-03

**Authors:** Yuma Uesaka, Kio Kawakatsu, Mana Akita, Toshiya Tsunakawa, Satoki Yoshida, Naoko Taki, Tiangao Jiang, Shanhu Liu, Eika W. Qian, Sho Usuki, Kazuya Nakata

**Affiliations:** 1 Graduate School of Bio-Applications and Systems Engineering, Tokyo University of Agriculture and Technology, 2-24-16 Naka-cho, 184-0012 Koganei, Tokyo Japan; 2 Henan Joint International Research Laboratory of Environmental Pollution Control Materials, Henan Key Laboratory of Polyoxometalate Chemistry, College of Chemistry and Chemical Engineering, Henan University, Kaifeng, 475004 PR China

**Keywords:** Methane formation, Photocatalysis, Glucose conversion, Co-catalysts, Methanation, Chemistry, Materials science

## Abstract

Sustainable production of CH_4_, an industrially important gas, from renewable resources presents a critical solution to energy security challenges. This study demonstrates photocatalytic conversion of glucose, a model biomass compound, to CH_4_ using a metal co-catalyst loaded TiO_2_ photocatalyst under ambient conditions. We confirmed that CH_4_ was formed through photocatalytic reduction of CO_2_, which was generated in situ during glucose oxidation, establishing a closed-loop conversion of biomass-derived carbon within a single reaction system. PtO_x_-TiO_2_ (x = 0, 1) exhibited significantly higher activity for CH_4_ production than PdO_x_-TiO_2_ (x = 0, 1). The CH_4_ yield with PtO_x_ loading was approximately ten times greater than that obtained with PdO_x_ loading, with an optimal PtO_x_ loading of 2.0 wt% yielding the highest CH_4_ amount of 10.600 µmol L^− 1^ after 6 h. In contrast, PdO_x_-TiO_2_ showed a higher selectivity for H_2_ generation. Analysis of the reaction products, including sugars (arabinose, erythrose, and glyceraldehyde) and organic acids (formic acid, acetic acid, and gluconic acid), elucidated the glucose degradation pathways. The mechanism of CH_4_ formation was identified as the methanation of CO_2_ and H^+^, both produced during photocatalytic oxidation of glucose and water. Deuterium-labeling experiments further revealed that the hydrogen atoms in CH_4_ originated from both glucose decomposition and water splitting. These findings demonstrate a novel and sustainable tandem photocatalytic process that integrates oxidation and reduction reactions on a single catalyst surface, providing mechanistic and practical insights into the direct conversion of biomass into CH_4_ under mild conditions.

**Supplementary Information:**

The online version contains supplementary material available at 10.1038/s41598-025-30321-w.

## Introduction

Biomass refers to organic materials derived from living organisms that can be used as renewable energy resources. Biomass possesses carbon-neutral characteristics that do not affect atmospheric CO_2_ concentrations^1,2^. Therefore, it has attracted attention as a climate change countermeasure^3,4^, and the utilization of biomass is being promoted globally toward the construction of energy supply processes that do not depend on fossil resources^5,6^. Furthermore, non-food biomass resources are attracting attention as they do not compete with food production^7,8^. Agricultural and forestry residues such as rice straw and thinned timber represent particularly promising examples of these resources^9,10^, and their effective utilization is expected to contribute to the realization of a sustainable circular society.

Since biomass is a sustainable resource, the production of useful substances through its conversion has attracted global attention^11–13^. Biomass primarily yields fuel and chemicals^14,15^. Fuels include bioethanol, which is produced by processes such as catalytic treatment under high-temperature and high-pressure conditions or microbial fermentation^16,17^. Biomass is also converted into useful chemicals, such as sugars^18,19^, which are in high demand as raw materials for pharmaceuticals and functional foods, and organic acids^15,20^, which possess high industrial utility value as basic chemicals. Biomass can be converted into a diverse range of useful substances, and conversion methods are being actively researched worldwide. Nevertheless, existing biomass conversion processes face numerous challenges, including the requirement of energy-intensive reaction conditions at high temperatures and pressures, significant environmental burdens, and high capital investment and operating costs^21–23^. Consequently, concerns have been raised regarding the sustainability of these conversion processes, and there is a demand for the development of novel biomass conversion processes that operate under milder conditions with reduced environmental impacts and enhanced economic viability.

In recent years, photocatalysis has attracted attention as a method of converting biomass into useful substances^24,25^. Photocatalysts are materials that absorb light, which is a renewable energy source, and promote oxidation and reduction reactions under mild conditions at ambient temperature and pressure^26^. When semiconductor photocatalysts are irradiated with light having energy greater than their band gap, electrons in the valence band are excited to the conduction band, and simultaneously, holes are generated in the valence band. These holes cause oxidation reactions, whereas excited electrons cause reduction reactions^26^. Furthermore, it is known that these oxidation and reduction reactions generate reactive oxygen species, such as hydroxyl radicals and superoxide anion radicals, from water and oxygen^27^.

The generation of various fuel sources and useful chemicals has been confirmed through photocatalytic biomass conversion. For example, Speltini et al. confirmed H_2_ generation by irradiating sunlight on cellulose suspensions containing Pt-loaded TiO_2_^28^, and Wang et al. converted C2-C6 compounds such as ethylene glycol, xylitol, and sorbitol, to methanol using Cu-loaded titanium oxide nanorods^29^. Furthermore, Paola et al. demonstrated vanillin production through ferulic acid conversion using TiO_2_ or TiO_2_-WO_3_^30^, and Nakata et al. confirmed the generation of sugars such as arabinose, erythrose, glyceraldehyde, and D-arabino-1,4-lactone through D-fructose decomposition using TiO_2_^31^.

Future fossil fuel shortages owing to the depletion of fossil resources have become a serious challenge^32,33^. This depletion necessitates the identification and development of alternative feedstocks for both energy production and chemical manufacturing. CH_4_ emerges as a particularly promising candidate due to its exceptional versatility across multiple industrial applications^34,35^. It functions not only as synthesis gas and fuel for power generation^36^, but also serves as a raw material for manufacturing basic chemicals such as ethylene and methanol^37,38^. This broad applicability positions CH_4_ as an industrially crucial gas that could help address the challenges posed by diminishing fossil fuel resources. Currently, CH_4_ production relies primarily on natural gas refining^39,40^. While this production method is considered low risk and economical^41,42^, it presents significant sustainability concerns given the ongoing depletion of fossil resources^43,44^. Based on the above background, in recent years, biogas upgrading and power-to-gas (PtG) technologies have attracted considerable attention from the perspective of reducing fossil resources consumption^45–47^. Biogas upgrading is a process to obtain high-purity CH_4_ by removing carbon dioxide (CO_2_), water vapor, hydrogen sulfide (H_2_S), and other impurities from biogas, a renewable resource^45,46^. However, this process involves complex purification steps, leading to high costs and significant energy consumption^48,49^. In contrast, PtG technology produces methane by electrolyzing water with renewable energy sources such as wind and solar power to generate H_2_, which is then reacted with CO_2_^45,47^. Nevertheless, this approach still faces critical challenges in terms of sustainability, since water electrolysis requires substantial energy input and the subsequent methanation reaction requires heating in the intermediate temperature range (300–400 °C)^49^.

From the perspective of sustainability, CO_2_ methanation has attracted attention^50,51^. This is a reaction that generally synthesizes CH_4_ by passing H_2_ and CO_2_ through catalysts under high temperatures and pressures^52,53^. While CO_2_ is typically emitted when CH_4_ is utilized, CO_2_ methanation recovers the emitted CO_2_ and synthesizes CH_4_ again, theoretically enabling carbon neutrality^54^. However, since the reaction must be conducted generally under mid-temperature (300–500 °C) and high-pressure, challenges include energy consumption and high risk^55^. Although recent studies have explored the reaction under low temperature (ca. 150 °C), such as photothermal methods^56,57^, the reaction still requires energy consumption for heating. In contrast, photocatalytic CO_2_ methanation utilizes renewable solar energy to convert CO_2_ and water into CH_4_ and has attracted significant attention as a sustainable pathway for CH_4_ production^58–60^. However, photocatalytic CO_2_ methanation still has low CH_4_ production efficiency and high cost for CO_2_ recovery and supply, and the design of high-pressure systems^60^. Based on the above background, this study focused on photocatalytic biomass conversion to establish a sustainable CH_4_ production process. Specifically, glucose was selected as a biomass derived compound, and CH_4_ generation via photocatalytic degradation was investigated, since the theoretical potential of glucose as a carbon source, which yields up to six CH_4_ molecules per glucose molecule.

Based on the above background, this study focused on photocatalytic biomass conversion to establish a sustainable CH_4_ production process. Specifically, glucose was selected as a biomass derived compound, and CH_4_ generation via photocatalytic degradation was investigated. Furthermore, to improve the photocatalytic activity, this study investigated the loading of metal co-catalysts, and PtO_x_ and PdO_x_ (x = 0, 1) were selected, as previous studies have reported that the loading of these co-catalysts is effective for CH_4_ production^61,62^. Using TiO_2_ loaded with these metal co-catalysts, the amount of CH_4_ generated by glucose degradation was compared and examined to determine the optimal metal co-catalyst for CH_4_ production. Additionally, elucidation of the CH_4_ generation mechanism was attempted through a detailed analysis of the glucose degradation pathways and product distribution in photocatalytic reactions.

## Experimental

### Preparation of metal co-catalyst-loaded TiO_2_

PtO_x_ and PdO_x_ particles were loaded on the TiO_2_ (here after, PtO_x_-TiO_2_ and PdO_x_-TiO_2_, respectively) surface using the photodeposition method^63^. Ultrapure water (50 mL) and 2-propanol (Wako Pure Chemical, 99.7%, 50 mL) were mixed and TiO_2_ (P25, Evonik) was added. Hexachloroplatinic (IV) acid hexahydrate (Wako Pure Chemical, 98.5%) or palladium (II) chloride (Wako Pure Chemical, 99.0%) was added as the metal precursor at 0.5 wt% of TiO_2_ based on the previous work^61^. The prepared suspension was stirred while being irradiated with ultraviolet (UV) light for 2 h using a 300 W HgXe lamp (UV-7, U-VIX). The light irradiation intensity was set at 6.0 mW cm^− 2^. After light irradiation, the suspension was filtered by suction and the obtained powder was washed with ultrapure water and dried at 110 °C for 12 h.

### Characterization of photocatalysts

The prepared photocatalysts were characterized using the following methods. To investigate the changes in the crystal structure of TiO_2_ caused by the loading of metal co-catalysts (PtO_x_ and PdO_x_), X-ray diffraction analysis was performed using an X-ray diffractometer (SmartLab, Rigaku). Measurements were conducted using Cu-Kα radiation with a tube voltage of 45 kV, tube current of 200 mA, scanning range of 10–60°, and scanning speed of 5.0° min^− 1^. X-ray photoelectron spectroscopy (XPS) analysis was performed using an X-ray photoelectron spectrometer (JPS9030, JEOL). Mg Kα radiation (tube voltage, 10 kV; tube current, 20 mA) was used as the X-ray source and charge correction was performed using the C 1s peak (285.0 eV) as a reference. H_2_-TPR was performed in a quartz U-tube and ca. 200 mg of sample was used in each measurement using an automated flow chemisorption analyzer (ChemBET Pulsar, Anton Paar). The samples were pretreated under He flow until the temperature was ramped to 150 °C at a rate of 20 °C min^− 1^. The flow of 5% H_2_/Ar was then switched into the system, and the sample was heated up to 800 °C at a rate of 5 °C min^− 1^. To confirm the microstructure of the metal co-catalysts on the TiO_2_ surface in detail, TEM images were obtained and the diameter measurements of PtO_x_ and PdO_x_ were performed for 20–30 particles using a 200 kV field-emission transmission electron microscope (JEM-2100 F, JEOL). Scanning Transmission Electron Microscope (STEM) images were obtained using a 200 kV schottky field-emission atomic-resolution scanning transmission electron microscope (JEM-ARM200F, JEOL). The specific surface area of the photocatalyst was determined by the Brunauer-Emmett-Teller (BET) method and was analyzed using a high-precision gas adsorption analyzer (BELSORP-mini II, Microtrac) after the sample was degassed in vacuum at 120 °C for 12 h. Diffuse reflectance spectra were measured using an UV-Vis spectrophotometer (UV-2600i, Shimadzu) under the following conditions: wavelength of 200–800 nm, slit width of 5.0 nm, low-speed scanning, and light source switching wavelength of 300 nm. The obtained spectra were processed using Kubelka-Munk transformation. Photoluminescence (PL) spectra of the photocatalysts were measured using a spectrofluorometer (JASCO, FP-6500). The measurement conditions were as follows: mode, emission; slit width, 5 nm; response, 2 s; sensitivity, low; excitation wavelength, 380 nm; starting wavelength, 350 nm; ending wavelength, 450 nm; data interval, 0.1 nm; and scan speed, 100 nm/min. Electrochemical impedance measurement (EIS) conditions were electrolyte: 0.1 mol L^− 1^ Na_2_SO_4_ (99.0%, Wako Pure Chemical Industries), reference electrode: Ag/AgCl electrode.

### Preparation of photoelectrode

The photoelectrode was fabricated using the following method. 30 mg of the powdered photocatalyst, 50 µL of 20 wt% Nafion solution (Wako Pure Chemical Industries), and 1 mL of ultrapure water were mixed and stirred for 30 min to prepare the photocatalyst suspension. 600 µL of this photocatalyst suspension was drop-casted onto fluorine-doped tin oxide (FTO, active area of ca. 2.25 cm^2^) coated glass, which were cleaned with ethanol (99.5%, Wako Pure Chemical Industries) before the casting, with an attached tape (1 cm width). The obtained sample was heat-treated at 120 °C for 1 h in a drying oven. The tape was removed, and a Tefloncoated Cu wire was soldered onto the exposed FTO part using a Cerasolzer (Kuroda Techno.). Subsequently, all parts exposed to Cerasolzer and FTO were coated with epoxy resin to create the photoelectrode.

### Glucose decomposition reaction

The reaction vessel contained a solution of glucose (Wako Pure Chemical Industries, 98.0%) dissolved in 20 mL of ultrapure water to achieve a concentration of 25 mmol L^− 1^, along with 20 mg of the photocatalyst. To maintain a constant reaction temperature, a cool plate was placed on a magnetic stirrer, and the reaction vessel was positioned on top. Prior to light irradiation, the mixture was stirred in the dark for 30 min and samples were collected from both the gas and liquid phases. A 300 W HgXe lamp (UV-7, U-VIX) was used as the light source, and the light intensity was set to 50 mW cm^− 2^. Gas samples (1 mL) were collected 3 h after the start of the light irradiation, and 1 mL of gas and 500 µL of liquid were collected after 6 h. The collected liquid samples were centrifuged, and the supernatant was filtered through a filter (RephiQuik Syringe Filter, pore size 0.22 μm, RephiLe Bioscience, Ltd.) for recovery.

Figure S1 and S2 represent a schematic overview and picture of the experimental setup respectively. In this study, a reactor was placed on a cool plate positioned above a magnetic stirrer. An aqueous glucose solution and photocatalyst were introduced into the reactor, and UV irradiation was applied to the reaction solution through a quartz glass window from the side. The gas chamber inside the reactor has a volume of ca. 5 mL.

### GC and HPLC analysis

The products obtained from glucose decomposition were analyzed as follows: quantitative analysis of CH_4_ and CO_2_ was conducted using a gas chromatograph (GC, GC2014, Shimadzu) with flame ionization detector (FID) detection connected to a methanizer (MTN-1, Shimadzu). A packed column (GC Stainless Column 4.0 m×3.0 mm I.D. Porapak Q 50/80, Shinwa Chemical Industries) was used with a column temperature of 40 °C, flow rate of 31 mL min^− 1^, detector temperature of 150 °C, and injection port temperature of 150 °C. Pure nitrogen was used as the carrier gas. Quantification of CH_4_ and CO_2_ in this study was performed as follows. First, 1 mL of the gas phase in the reaction vessel was sampled and injected into the GC for analysis. Based on the obtained peak areas, the concentrations of the target gases in 1 mL of the gas phase (ppm) were determined using the single-point calibration method. As standard samples, push-can type standard gases of methane (GL Sciences, 99.9%), and carbon dioxide (GL Sciences, 99.9%) were used. Subsequently, from the calculated ppm values, the volume (mL) of each target gas contained in 1 mL of the gas phase was obtained, and the amount of gas produced was calculated by dividing this volume by the standard molar volume of an ideal gas (22.4 × 10^3^ mL mol^− 1^).

The quantitative analysis of H_2_ was performed using a GC (GC2014, Shimadzu) with a thermal conductivity detector (TCD). A packed column (GC Stainless Column 2.0 m×3.0 mm I.D. Molecular Sieve 13 × 60/80, Shinwa Chemical Industries) was used, with the column temperature set to 50 °C, flow rate to 10 mL min^− 1^, detector temperature to 100 °C, and injection port temperature to 70 °C. Pure argon was used as the carrier gas. Quantification of H_2_ this study was performed in the same method for CH_4_ and CO_2_. As standard samples, push-can type standard gases of hydrogen (GL Sciences, 99.99%) were used.

The starting material, glucose, and other sugars produced after the reaction were labeled with 4-aminobenzoic acid ethyl ester (ABEE, Tokyo Chemical Industry) and then subjected to qualitative and quantitative analysis using high performance liquid chromatography (HPLC, Shimadzu). ABEE labeling was performed according to the following procedure: First, ABEE, sodium cyanoborohydride (Tokyo Chemical Industry), and acetic acid (Wako Pure Chemical Industries) were mixed at a molar ratio of 8:2:27, and the ABEE labeling reagent was prepared by dissolving the mixture in methanol (Wako Pure Chemical Industries) equivalent to 9 times the total volume of the three substances contained in the mixture. To 40 µL of ABEE labeling reagent, 10 µL of the sample was added and stirred for 30 s. Subsequently, centrifugation was performed at 10,000×g for 2 min using a centrifuge (3780, KUBOTA). The solution was placed in a block bath (DB105, SCINICS) and incubated at 80 °C for 1 h. After incubation, the sample was removed and air cooled for 2 min to return to room temperature. Subsequently, centrifugation was performed at 10,000×g for 2 min using a centrifuge. Ultrapure water (200 µL) and chloroform (200 µL; Wako Pure Chemical Industries) were added to the sample, stirred for 1 min, and centrifuged again under the same conditions. From the solution separated into two layers, 150 µL of the supernatant was collected, diluted with 300 µL of ultrapure water, and centrifuged under the same conditions. This sample was used for HPLC measurement. A reverse-phase column (CAPCELL PAK C18 AQ, column 150 × 4.6 mm I.D., OSAKA SODA) was used, and a wavelength of 305 nm was detected using a UV detector (SPD-20 A, Shimadzu). The column temperature was set to 40 °C, flow rate was 1.0 mL min^− 1^, injection volume was 20 µL, and elution time was 60 min. The carrier solution used was a mixture of 20 mmol L^− 1^ ammonium acetate aqueous solution (Wako Pure Chemical Industries) and acetonitrile (Wako Pure Chemical Industries) at a volume ratio of 87:13. For gluconic acid, formic acid, and acetic acid quantification, HPLC (Shimadzu) was used with an organic acid analysis column (Rezex ROA-Organic Acid, 300 mm×7.8 mm I.D., Phenomenex) and UV detection at 210 nm. A 2.5 mmol L^− 1^ sulfuric acid solution was used as the mobile phase with a flow rate of 0.5 mL min^− 1^ at a column temperature of 60 °C.

### Deuterium labeling experiments

To analyze the reaction mechanism through deuterium labeling, photocatalytic reactions were conducted using D_2_O instead of ultrapure water. In the reaction vessel, 20 mg of PtO_x_-TiO_2_ with a PtO_x_ loading rate of 0.5 wt% relative to TiO_2_ was added, and 20 mL of D_2_O was added. The reaction was performed in a closed system, using a lid equipped with a septum for gas sampling. UV light (light irradiation intensity: 50 mW cm^− 2^) was irradiated using a HgXe lamp (UV-7, U-VIX). After stirring in the dark for 30 min, the UV irradiation was initiated. Gas samples (1 mL) were collected from the gas phase 3 and 6 h after the start of the reaction, and molecular weight analysis of CH_4_ was performed using GC-MS (GCMSQP2020, Shimadzu). RT-Msieve 5 A (RESTEK) was used as the separation column. The column flow rate was 32.9 mL min^− 1^, the column oven temperature was 30 °C, the vaporization chamber temperature was 150 °C, the interface temperature was 190 °C, the detector voltage was 0.1 kV, the split ratio was 20, and *m/z* was set to 16, 17, 18, 19, and 20.

### CH_4_ generation from CO_2_ aqueous solution

To verify the hypothesis that CH_4_ generation by the photocatalytic reaction originates from methanation, which is the reaction between CO_2_ and H^+^, CH_4_ generation experiments using CO_2_ aqueous solutions were conducted. To maintain a constant reaction temperature, a cool plate was placed on a magnetic stirrer, and the reaction vessel was positioned on top. Ultrapure water (20 mL) and the photocatalyst (20 mg) were added to the sealed reaction vessel. Before starting the light irradiation, CO_2_ bubbling was performed for 30 min on the solution in the reaction vessel. Subsequently, stirring was conducted in the dark, and the gas samples were collected. A 300 W HgXe lamp (UV-7, U-VIX) was used as the light source, and the light irradiation intensity was set to 50 mW cm^− 2^. Gas samples (1 mL each) were collected 3 and 6 h after the start of light irradiation.

### Catalytic reaction of CO_2_ and H^+^ in the dark

To confirm whether the CH_4_ generated from CO_2_ and H^+^ could be produced by a catalytic reaction in the dark as a control, the following experiment was conducted. A cool plate was installed on a magnetic stirrer to maintain a constant reaction temperature, and the reaction vessel was placed on top. The reaction vessel was charged with 20 mL of ultrapure water containing 25 mmol L^− 1^ glucose and 20 mg of the photocatalyst. The reaction vessel was sealed with a lid for gas sampling. Pure hydrogen (99.99%) and pure CO_2_ (99.9%) were added to the reaction vessel in 50 µL volumes, and 1.0 mL of gas from within the reaction vessel was sampled for quantitative analysis before addition, 3 h after addition, and 6 h after addition in the dark.

Quantitative analysis of CH_4_ was performed using a gas chromatograph (GC2014, Shimadzu) equipped with a flame ionization detector (FID). A packed column (GC Stainless Column 4.0 m × 3.0 mm I.D. Porapak Q 50/80, Shinwa Chemical Industries) was used with a column temperature of 40 °C, flow rate of 31 mL min^− 1^, detector temperature of 150 °C, and injection port temperature of 150 °C. Pure nitrogen was used as the carrier gas.

## Results and discussion

### Characterization of PtO_x_ and PdO_x_

**Fig. 1:**
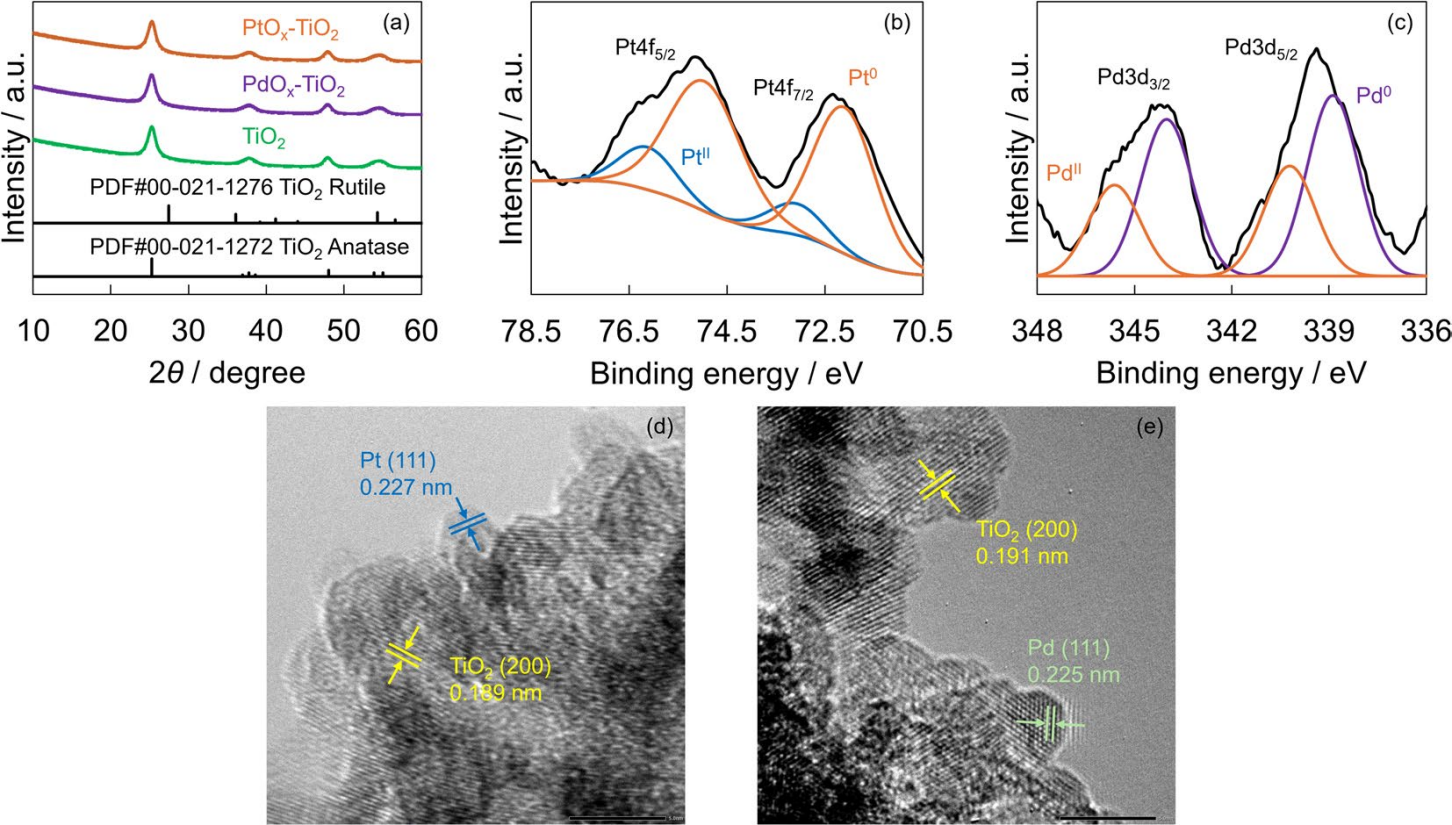
(**a**) XRD patterns of the prepared photocatalysts (orange: PtO_x_-TiO_2_; purple: PdO_x_-TiO_2_; green: TiO_2_). (**b**) XPS spectrum of PtO_x_-TiO_2_ (Pt 4f orbital, orange: metallic Pt, blue: PtO). (**c**) XPS spectrum of PdO_x_-TiO_2_ (Pd 3d orbital, purple: metallic Pd, orange: PdO). (**d**) TEM image of the PtO_x_-TiO_2_ photocatalyst. (**e**) TEM image of the PdO_x_-TiO_2_ photocatalyst.

Figure 1a shows the X-ray diffraction patterns of the prepared PtO_x_-TiO_2_, PdO_x_-TiO_2_, and pristine TiO_2_. The peak at 25.3° for TiO_2_ corresponds to the (101) plane of the anatase phase, whereas the peak at 37.8° is assigned to the (103), (004), and (112) planes of the anatase phase. Additionally, the peak at 47.9° corresponds to the (200) plane of the anatase phase^64,65^. The peak at 54.7° was associated with the (105) and (211) planes of the anatase phase and the (211) plane of the rutile phase^64,65^. Since these peaks were similarly observed in PtO_x_- and PdO_x_-loaded TiO_2_, no significant changes were observed in the X-ray diffraction patterns of TiO_2_ due to PtO_x_ and PdO_x_ loading.

To analyze the chemical states of PtO_x_-TiO_2_ and PdO_x_-TiO_2_, XPS analysis was conducted. Figure 1b shows the XPS spectrum of the Pt 4f orbital in PtO_x_-TiO_2_, while Fig. 1c displays the corresponding Pd 3d orbital spectrum in PdO_x_-TiO_2_. In the Pt 4f spectrum, two main peaks (Pt 4f_5/2_ and Pt 4f_7/2_) are observed. The peaks at 72.1 eV and 75.0 eV are attributed to metallic Pt, while those at 73.1 eV and 76.1 eV correspond to PtO^66^. Similarly, the Pd 3d spectrum exhibits two main peaks (Pd 3d_3/2_ and Pd 3d_5/2_), with peaks at 338.9 eV and 345.6 eV attributed to metallic Pd, and peaks at 340.2 eV and 345.6 eV assigned to PdO^67^. These XPS results confirm the successful loading of both metallic and oxidized forms of Pt and Pd onto the TiO_2_ surface. These XPS results confirm the successful loading of both metallic and oxidized forms of Pt and Pd onto the TiO_2_ surface. However, the XPS spectrum suggested that the relative abundance of oxidized species (PtO, PdO) remained lower than that of the metallic species (Pt^0^, Pd^0^). The deposition of the oxides may derive from the partial oxidation of Pt^0^ or Pd^0^ by photogenerated holes (Eqs. 1 and 2) or dissolved oxygen (Eqs. 3 and 4):

1 $$Pt^{0} + {\text{ }}2h^{ + } + {\text{ }}H_{2} O{\text{ }} \to {\text{ }}PtO{\text{ }} + {\text{ }}2H^{ + }$$

2 $$Pt^{0} + {\text{ }}2h^{ + } + {\text{ }}H_{2} O{\text{ }} \to {\text{ }}PdO{\text{ }} + {\text{ }}2H^{ + }$$

3 $$Pt^{0} + {\text{ }}1/2O_{2} \to {\text{ }}PtO$$

4 $$Pt^{0} + {\text{ }}1/2O_{2} \to {\text{ }}PdO$$

Although these oxidation processes are thermodynamically possible, their occurrence is significantly suppressed due to the presence of 2-propanol as a hole scavenger, which minimizes the oxidation of Pt^0^ and Pd^0^ via the photodeposition. Consequently, the relative abundance of oxidized species (PtO, PdO) remains lower than that of metallic Pt and Pd in the final catalysts.

Figure S3 shows the H_2_-TPR profiles of the prepared PtO_x_-TiO_2_ and pristine TiO_2_. A peak was observed for PtO_x_-TiO_2_ at approximately 60–90 °C, which can be attributed to PtO_x_^68^. Figure S4 also shows the H_2_-TPR profiles of the prepared PdO_x_-TiO_2_ and TiO_2_. A peak was observed for PdO_x_-TiO_2_ at approximately 60–70 °C, which can originate from PdO_x_^69^.

TEM measurements were conducted to observe the average size and crystal structure of PtO_x_ and PdO_x_ nanoparticles loaded on TiO_2_. Figure 1d shows the TEM image of PtO_x_-TiO_2_. The TEM image revealed that Pt nanoparticles with an average diameter of approximately 3 nm were attached to the surface of the TiO_2_ particles. The measurement of the lattice fringe spacings confirmed periodic structures of 0.189 nm and 0.227 nm, which correspond to the (200) plane of TiO_2_ and the (111) plane of Pt, respectively. Similarly, Fig. 1e shows the TEM image of PdO_x_-TiO_2_, which revealed that Pd nanoparticles with an average diameter of approximately 4 nm were attached to the surface of the TiO_2_ particles. The lattice fringe spacings of 0.191 and 0.225 nm correspond to the (200) plane of TiO_2_ and the (111) plane of Pd, respectively. Notably, the lattice fringes attributed to PtO and PdO were not observed in either case. These observations suggest that the loading amounts of PtO and PdO were lower than their respective metallic forms, which is consistent with the XPS results.

Figure S5 and S6 shows the PtO_x_ and PdO_x_ particle size distributions for PtO_x_-TiO_2_ and PdO_x_-TiO_2_, respectively. The mean particle size of the PtO_x_ particles was determined to be 3.38 ± 0.71 nm. Particles with a diameter of 2 nm accounted for 4.2% of the total, while those of 3 nm, 4 nm, and 5 nm represented 62.5%, 25.0%, and 8.3%, respectively. No particles with a diameter of 1 nm were observed. The mean particle size of the PdO_x_ particles was determined to be 3.86 ± 0.47 nm. Particles with a diameter of 3 nm accounted for 16.7% of the total, while those of 4 nm, and 5 nm represented 70.8%, and 4.2%, respectively. No particles with a diameter of 1 nm and 2 nm were observed.

STEM measurements were conducted to observe photo-induced changes in the oxidation state of PtO_x_ particles in PtO_x_-TiO_2_ during the glucose decomposition reaction. STEM images of PtO_x_-TiO_2_ before and after 6 h of light irradiation are shown in Figures S7 and S8, respectively. The bright spots in Figure S7 and the dark spots in Figure S8 are both attributed to PtO_x_ particles. These observations indicate that the PtO_x_ particles changed from bright to dark during light irradiation. This contrast change may be attributed to the oxidation of Pt to PtO during light irradiation, or to the coverage of the particles by glucose and its degradation byproducts.

Figure S9 shows the UV-vis spectra of PtO_x_-TiO_2_, PdO_x_-TiO_2_, and bare TiO_2_. In the wavelength range of 400–800 nm, both PtO_x_-TiO_2_ and PdO_x_-TiO_2_ exhibit higher apparent absorption than bare TiO_2_. Although a localized surface plasmon resonance (LSPR) of Pt or Pd has been reported around 410 nm^70^, our spectra do not show a distinct, narrow plasmon band; instead, the enhancement is broad across the visible region. Considering that TEM reveals sub-10 nm particles for PdO_x_-TiO_2_ in this study (Fig. 1e) and that Pd particles below 10 nm typically exhibit only UV features^71^, together with the presence of both metallic and oxidized states of Pt and Pd confirmed by XPS (Fig. 1b, c), it is likely that partial oxidation of Pt and Pd dampens the plasmonic response. Therefore, while some contribution from LSPR cannot be completely excluded, it is not considered the dominant origin of the enhanced visible absorption. Instead, the increased visible absorption is attributed to a combination of (i) interfacial charge-transfer transitions between supported PtO_x_ or PdO_x_ species and TiO_2_, (ii) defect-related sub-band-gap absorption arising from Ti^3+^ centers and oxygen vacancies generated during photodeposition, (iii) enhanced light scattering by supported nanoparticles that increases the effective optical path length in diffuse-reflectance measurements, and (iv) the intrinsic visible-light absorption of PtO and PdO^72,73^. These factors collectively account for the broadband enhancement observed for both PtO_x_-TiO_2_ and PdO_x_-TiO_2_.

### Glucose conversion and products analyses

**Fig. 2:**
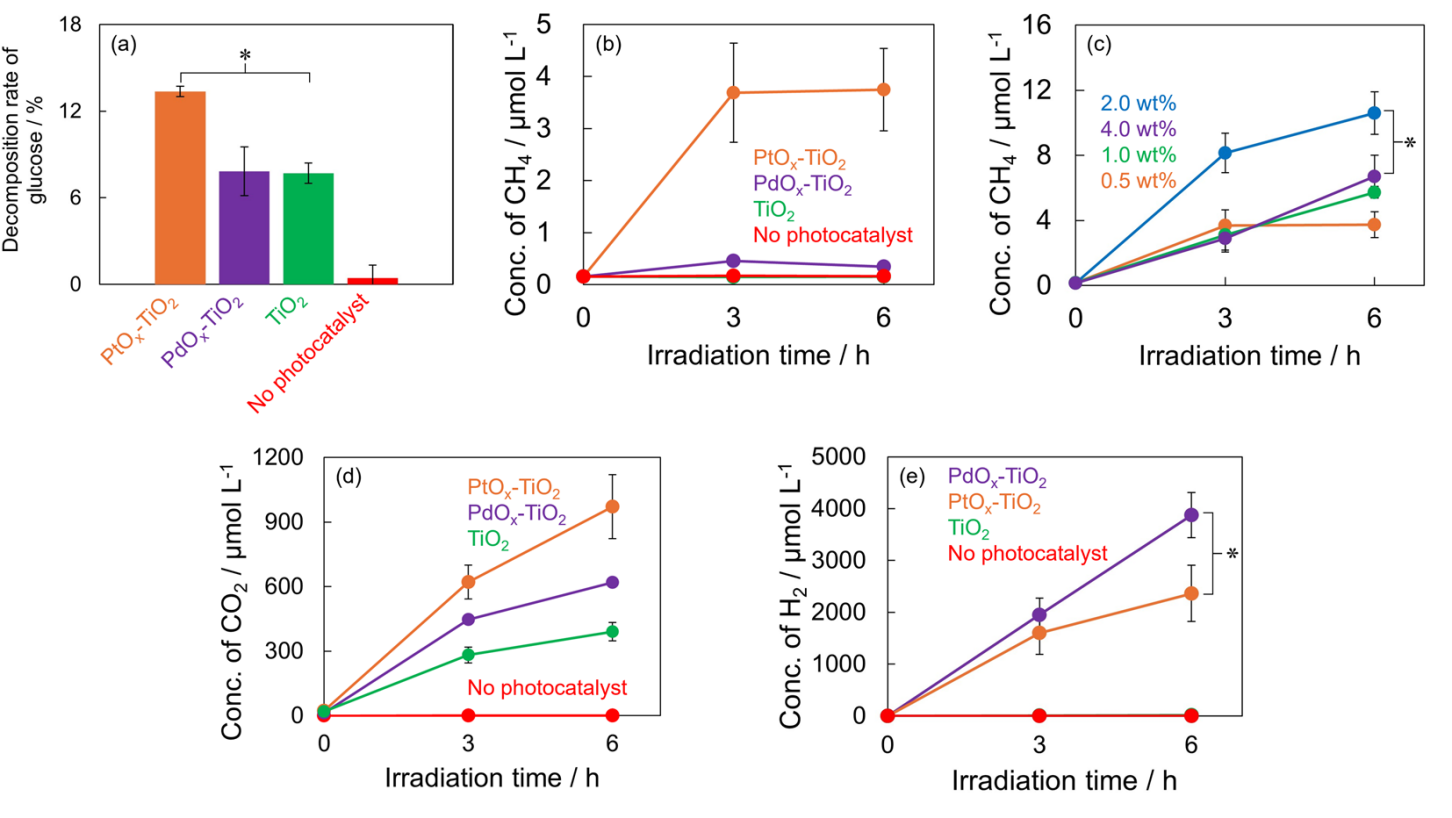
(**a**) Decomposition rate of glucose after 6 h of the experiment (*n* = 3, *: *p* < 0.05, orange: PtO_x_-TiO_2_, purple: PdO_x_-TiO_2_, green: TiO_2_, red: No photocatalyst). (**b**) Changes in CH_4_ concentration (*n* = 3, orange: PtO_x_-TiO_2_, purple: PdO_x_-TiO_2_, green: TiO_2_, red: no photocatalyst). (**c**) Time course of CH_4_ concentration using PtO_x_-TiO_2_ with different PtO_x_ loading rates on TiO_2_ (*n* = 3, *: *p* < 0.05, orange: 0.5 wt%, green: 1.0 wt%, blue: 2.0 wt%, purple: 4.0 wt%). Time course of (**d**) CO_2_, and (**e**) H_2_ concentration (*n* = 3, *: *p* < 0.05, orange: PtO_x_-TiO_2_, purple: PdO_x_-TiO_2_, green: TiO_2_, red: No photocatalyst).

Figure 2a shows the glucose decomposition rates using the prepared PtO_x_-TiO_2_ and PdO_x_-TiO_2_, as well as control experiments using TiO_2_ alone, and without the photocatalyst. The glucose decomposition rate was calculated using the formula (Glc_0_-Glc_6_)/Glc_0_ × 100, where Glc_0_ represents the glucose concentration before light irradiation and Glc_6_ represents the glucose concentration after 6 h of light irradiation. The glucose decomposition rates were 13.36% for PtO_x_-TiO_2_, 7.83% for PdO_x_-TiO_2_, 7.70% for TiO_2_, and 0.44% for the case without photocatalyst. The glucose decomposition rate was improved by PtO_x_ loading compared to that of TiO_2_, whereas no significant difference was observed between PdO_x_-TiO_2_ and TiO_2_.

Figure 2b shows the changes in CH_4_ concentration when using PtO_x_-TiO_2_, PdO_x_-TiO_2_, TiO_2_, and without the photocatalyst. In the case of PtO_x_-TiO_2_, CH_4_ was generated at 3.691 µmol L^− 1^ after 3 h, and the concentration was approximately the same at 3.748 µmol L^− 1^ after 6 h. In contrast, with PdO_x_-TiO_2_, CH_4_ concentration was minimal at 0.456 µmol L^− 1^ after 3 h and 0.349 µmol L^− 1^ after 6 h. CH_4_ concentration was not detected when TiO_2_ was used alone or without a photocatalyst.

PtO_x_-TiO_2_ showed significant CH_4_ generation compared to PdO_x_-TiO_2_ and pristine TiO_2_. Therefore, the effect of PtO_x_ loading ratio on TiO_2_ was investigated to identify the optimal photocatalytic conditions for CH_4_ generation from glucose. Figure 2c shows the changes in CH_4_ concentration when using PtO_x_-TiO_2_ with different loading ratios was used. In the system using PtO_x_-TiO_2_, the CH_4_ generation after 6 h of light irradiation was 3.748 µmol L^− 1^ when the PtO_x_ loading ratio on TiO_2_ was 0.5 wt%, 5.732 µmol L^− 1^ at 1.0 wt%, 10.600 µmol L^− 1^ at 2.0 wt%, and 6.696 µmol L^− 1^ at 4.0 wt%. These results revealed that maximum CH_4_ concentration was achieved when using PtO_x_-TiO_2_ with a loading ratio of 2.0 wt%.

STEM measurements were conducted to clarify the underling mechanism that the CH_4_ production rate peaked at 2.0 wt% PtO_x_ loading but declined at 4.0 wt%. Figures S10 and S11 show the STEM images of 2.0 wt% PtO_x_-TiO_2_ and 4.0 wt% PtO_x_-TiO_2_, respectively. In the 2.0 wt% loading system, uniformly sized PtO_x_ nanoparticles with an average diameter of approximately 7 nm were observed to be dispersed on the TiO_2_ surface. On the other hand, in the 4.0 wt% loading system, aggregated PtO_x_ particles (average size: 15 nm) were observed, indicating a significant deterioration in dispersion.

The decline in photocatalytic activity at these loading rates can be attributed to two possible factors: (i) the reduction of the active site accessibility and light-harvesting efficiency and (ii) the decrease in the metal-semiconductor interfacial area due to nanoparticle aggregation. To assess the contribution of the decreased accessible active sites to the overall photocatalytic performance, the accessible active site density of 2.0 wt% PtO_x_-TiO_2_ and 4.0 wt% PtO_x_-TiO_2_ was calculated as follows. The accessible active site density for 2.0 wt% PtO_x_-TiO_2_ and 4.0 wt% PtO_x_-TiO_2_ was estimated according to the following procedure. First, the specific surface area of TiO_2_ (P25, Evonik) used in this study was determined to be 49.2 m^2^ g^− 1^ by BET method. Therefore, the theoretical total surface area of TiO_2_ weighing x g can be calculated as 4.92 × 10^5^x cm^2^. In the case of 2.0 wt% PtO_x_-TiO_2_, x g of TiO_2_ theoretically supports 0.02x g of PtO_x_, while 4.0 wt% PtO_x_-TiO_2_ supports 0.04x g of PtO_x_. STEM analysis (Figures S10 and S11) revealed that the average diameters of PtO_x_ particles in the 2.0 wt% and 4.0 wt% loading samples were approximately 7 nm and 15 nm, respectively. Assuming that each PtO_x_ particle is a perfect sphere, the corresponding particle volumes were calculated to be 5.72π × 10^− 20^ cm^3^ for 2.0 wt% and 5.63π × 10^− 19^ cm^3^ for 4.0 wt%. For simplicity, if the PtO_x_ particles are considered to consist entirely of metallic Pt with a density of 21.45 g cm^− 3^, the mass of a single particle can be estimated as 1.23π × 10^− 18^ g and 1.21π × 10^− 17^ g for the 2.0 wt% and 4.0 wt% systems, respectively. Based on these values, the total number of particles corresponding to 0.02x g and 0.04x g of Pt loading can be derived. Treating the photocatalyst as a flat surface, each PtO_x_ particle was approximated as a circle, with a projected area of 1.23π × 10^− 13^ cm^2^ for 2.0 wt% and 5.63π × 10^− 13^ cm^2^ for 4.0 wt%. Consequently, the theoretical total surface area of PtO_x_ particles was estimated to be 2.00 × 10^3^x cm^2^ for the 2.0 wt% sample and 1.86 × 10^3^x cm^2^ for the 4.0 wt% sample. From these results, the theoretical accessible active site densities were calculated to be 99.59% and 99.62% for the 2.0 wt% and 4.0 wt% systems, respectively, as shown in Eqs. 5 and 6.

5 $$1{\text{ }} - {\text{ }}\left( {2.00{\text{ }} \times {\text{ }}10^{3} x{\text{ }}/{\text{ }}4.92{\text{ }} \times {\text{ }}10^{5} x} \right){\text{ }} = {\text{ }}99.59$$

6 $$1{\text{ }} - {\text{ }}\left( {1.86{\text{ }} \times {\text{ }}10^{3} x{\text{ }}/{\text{ }}4.92{\text{ }} \times {\text{ }}10^{5} x} \right){\text{ }} = {\text{ }}99.62$$

These calculations indicate that the accessible active site density was slightly higher in the 4.0 wt% PtO_x_ loading system. Thus, the observed difference in photocatalytic activity between the two samples is unlikely to result from variations in light-shielding by the PtO_x_ nanoparticles. Instead, it is more likely attributable to a reduction in the effective metal-semiconductor interfacial area caused by nanoparticle aggregation.

Figure 2d and e show the photocatalytic generation of CO_2_ and H_2_ from aqueous glucose solutions using PtO_x_-TiO_2_, PdO_x_-TiO_2_, and TiO_2_ as photocatalysts, compared to light irradiation without photocatalyst addition. For CO_2_ generation, the concentration after 6 h of light irradiation was 971 µmol L^− 1^ for PtO_x_-TiO_2_, 620 µmol L^− 1^ for PdO_x_-TiO_2_, and 391 µmol L^− 1^ for TiO_2_. In contrast, glucose photolysis without a photocatalyst produced only 0.110 µmol L^− 1^ of CO_2_. The CO_2_ generation performance followed the order PtO_x_-TiO_2_ > PdO_x_-TiO_2_ > TiO_2_. For H_2_ generation, the concentration after 6 h of light irradiation reached 3874 µmol L^− 1^ for PdO_x_-TiO_2_, 2365 µmol L^− 1^ for PtO_x_-TiO_2_, and 15.3 µmol L^− 1^ for TiO_2_. No H_2_ generation was observed without the photocatalyst. The H_2_ generation performance demonstrated the order PdO_x_-TiO_2_ > PtO_x_-TiO_2_ > TiO_2_.

These results reveal distinct catalytic selectivity patterns among the photocatalysts. While PtO_x_-TiO_2_ exhibited superior CO_2_ generation efficiency, PdO_x_-TiO_2_ demonstrated the highest H_2_ production capability. Both metal co-catalyst modified TiO_2_ significantly outperformed pure TiO_2_ for both products, confirming the enhancement effect of the metal co-catalysts on the photocatalytic glucose conversion process.

It has been reported that photocatalytic decomposition of glucose produces sugars^74^. The analysis of these sugar products is important for understanding the reaction pathways.

**Fig. 3:**
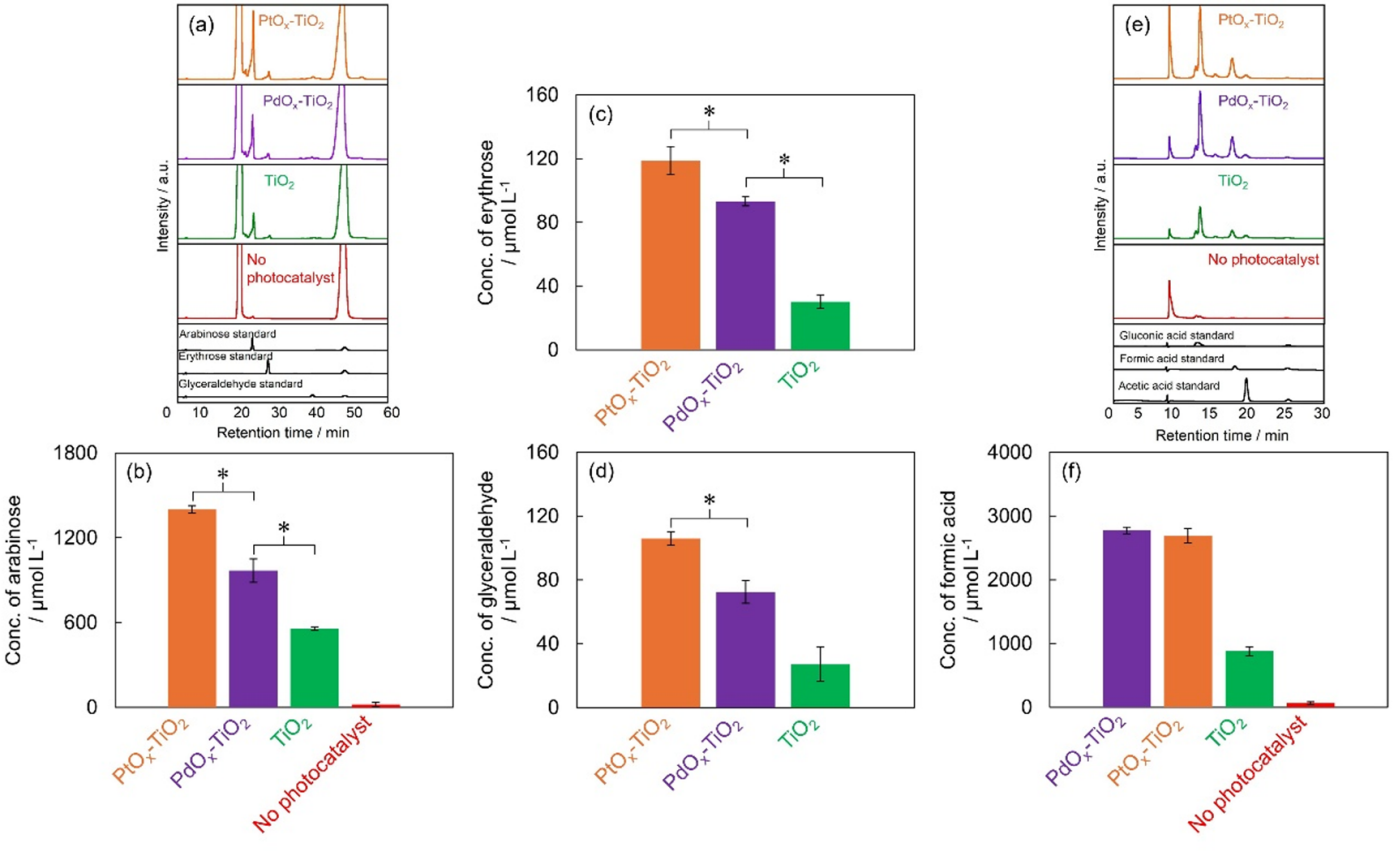
(**a**) HPLC chromatograms of sugar products and standards of arabinose, erythrose and glyceraldehyde. Concentration of (**b**) arabinose, (**c**) erythrose, and (**d**) glyceraldehyde in the products (*n* = 3, *: *p* < 0.05). (**e**) HPLC chromatograms of organic acid products and standards of gluconic acid, acetic acid and formic acid. (**f**) Concentration of formic acid in the products. All products obtained from glucose decomposition after 6 h of light irradiation using PtO_x_-TiO_2_ (orange), PdO_x_-TiO_2_ (purple), and TiO_2_ (green), no photocatalyst (red).

Figure 3a shows the HPLC chromatograms of the sugar products contained in the solution after glucose decomposition for 6 h using photocatalysts PtO_x_-TiO_2_, PdO_x_-TiO_2_, and TiO_2_, as well as the results from a control experiment involving glucose decomposition under light irradiation alone without photocatalysts. Sugars contained in each sample were identified by comparing the retention times (R.T.) of sugar standard samples after ABEE labeling. The R.T. of standard substances were approximately 21.4 min for arabinose, 25.8 min for erythrose, and 38.8 min for glyceraldehyde, respectively. Based on a comparison with the R.T. of standard substances, peaks corresponding to arabinose, erythrose, and glyceraldehyde were clearly detected in all photocatalytic systems using PtO_x_-TiO_2_, PdO_x_-TiO_2_, and TiO_2_, confirming the formation of these sugar compounds. In contrast, in the photolysis of glucose alone without photocatalysts, only the formation of arabinose was detected, and its peak was confirmed in the chromatogram, while the peaks of erythrose and glyceraldehyde were below the detection limit.

Figures 3b-d present the concentrations of sugar products in the reaction solution after 6 h of photocatalytic glucose decomposition. Arabinose concentrations (Fig. 3b) were 1401 µmol L^− 1^ for PtO_x_-TiO_2_, 968 µmol L^− 1^ for PdO_x_-TiO_2_, 556 µmol L^− 1^ for TiO_2_, and 19 µmol L^− 1^ for glucose decomposition without photocatalyst. Erythrose concentrations (Fig. 3c) were 119 µmol L^− 1^ for PtO_x_-TiO_2_, 93 µmol L^− 1^ for PdO_x_-TiO_2_ and 30 µmol L^− 1^ for TiO_2_. Glyceraldehyde concentrations (Fig. 3d) were 106 µmol L^− 1^ for PtO_x_-TiO_2_, 72 µmol L^− 1^ for PdO_x_-TiO_2_, and 27 µmol L^− 1^ for TiO_2_. The concentration of all three sugar products decreased in the order of PtO_x_-TiO_2_ > PdO_x_-TiO_2_ > TiO_2_, confirming that metal co-catalyst loading significantly enhanced sugar formation from glucose decomposition, and PtO_x_-TiO_2_ exhibited the highest glucose decomposition rate and the maximum production amounts for all sugar compounds. This result suggests that the PtO_x_ loading promotes the photocatalytic reaction. On the other hand, although PdO_x_ loading did not cause significant improvement in the glucose decomposition rate itself, the production amounts of arabinose, erythrose, and glyceraldehyde were clearly improved. This indicates that PdO_x_-TiO_2_ possesses a specific reaction selectivity. Thus, PdO_x_ loading guided the photocatalytic activity of TiO_2_ to specific reaction pathways, thereby promoting the conversion of glucose to these sugar compounds.

Organic acids are generated during the photocatalytic degradation of glucose. Figure 3e shows HPLC chromatograms of organic acids in glucose degradation sample using PtO_x_-TiO_2_, PdO_x_-TiO_2_, and TiO_2_, as well as in glucose degradation without a photocatalyst, under light irradiation. The R.T. of standards were approximately 11.9 min for gluconic acid, approximately 16.9 min for formic acid, and approximately 18.8 min for acetic acid, respectively. By comparing the retention times of reaction products with those of standard compounds, the formation of gluconic acid, formic acid, and acetic acid was confirmed in all photocatalytic systems. Furthermore, the formation of these acids was also confirmed in the glucose degradation without photocatalyst.

Figure 3f shows a comparison of the formic acid concentrations produced by the different photocatalysts. The formic acid concentrations were 2691 µmol L^− 1^ for PtO_x_-TiO_2_, 2775 µmol L^− 1^ for PdO_x_-TiO_2_, 881 µmol L^− 1^ for TiO_2_, and 63 µmol L^− 1^ in the absence of photocatalyst. No significant difference was observed between the formic acid concentrations of PtO_x_-TiO_2_ and PdO_x_-TiO_2_.

Based on the aforementioned results, it was demonstrated that the metal co-catalysts loading onto TiO_2_ contributed significantly to the enhancement of formic acid production. Notably, the observation that PdO_x_-TiO_2_ exhibited the highest formic acid concentration suggests that the formic acid generation pathway proceeds preferentially in PdO_x_-loaded systems. Conversely, for sugar production (arabinose, erythrose, glyceraldehyde), PtO_x_-TiO_2_ demonstrated the highest production. Initially, it was anticipated that the superior oxidative capability of PtO_x_-TiO_2_ would confer advantages in formic acid generation as well, however, the experimental findings deviated from this expectation. This phenomenon is attributed to the efficient secondary oxidation of formic acid within the PtO_x_-TiO_2_ system. Specifically, it is postulated that the subsequent oxidative conversion of generated formic acid to CO_2_ is substantially enhanced in PtO_x_-TiO_2_ systems. This hypothesis is corroborated by the maximum CO_2_ production observed in the PtO_x_-TiO_2_ system, demonstrating internal consistency with the proposed mechanism.

Table S1 represents the carbon balance after 6 h of light irradiation. The carbon balance of each carbon-containing compound with a known production amount was calculated, and an approximate total carbon value was obtained by summing these values. The carbon balances were 94.69% for PtO_x_-TiO_2_, 98.52% for PdO_x_-TiO_2_, 95.18% for TiO_2_, and 99.69% for the case without photocatalyst. All of these values were close to 100% and the remaining fraction, less than 100%, is considered to be composed of other organic compounds that were not quantified in this study.

### Discussion of mechanism of CH_4_ generation

Glucose addition is essential for CH_4_ generation, as demonstrated by a control experiment without glucose. Figure S12 shows the CH_4_ concentrations with and without glucose addition. After 6 h of light irradiation, the CH_4_ concentration for glucose solution decomposition was 3.748 µmol L^− 1^, whereas only 0.240 µmol L^− 1^ was observed in the absence of glucose. The significantly reduced CH_4_ production without glucose compared to that with glucose solution decomposition confirms that glucose introduction is essential for efficient CH_4_ generation.

**Fig. 4:**
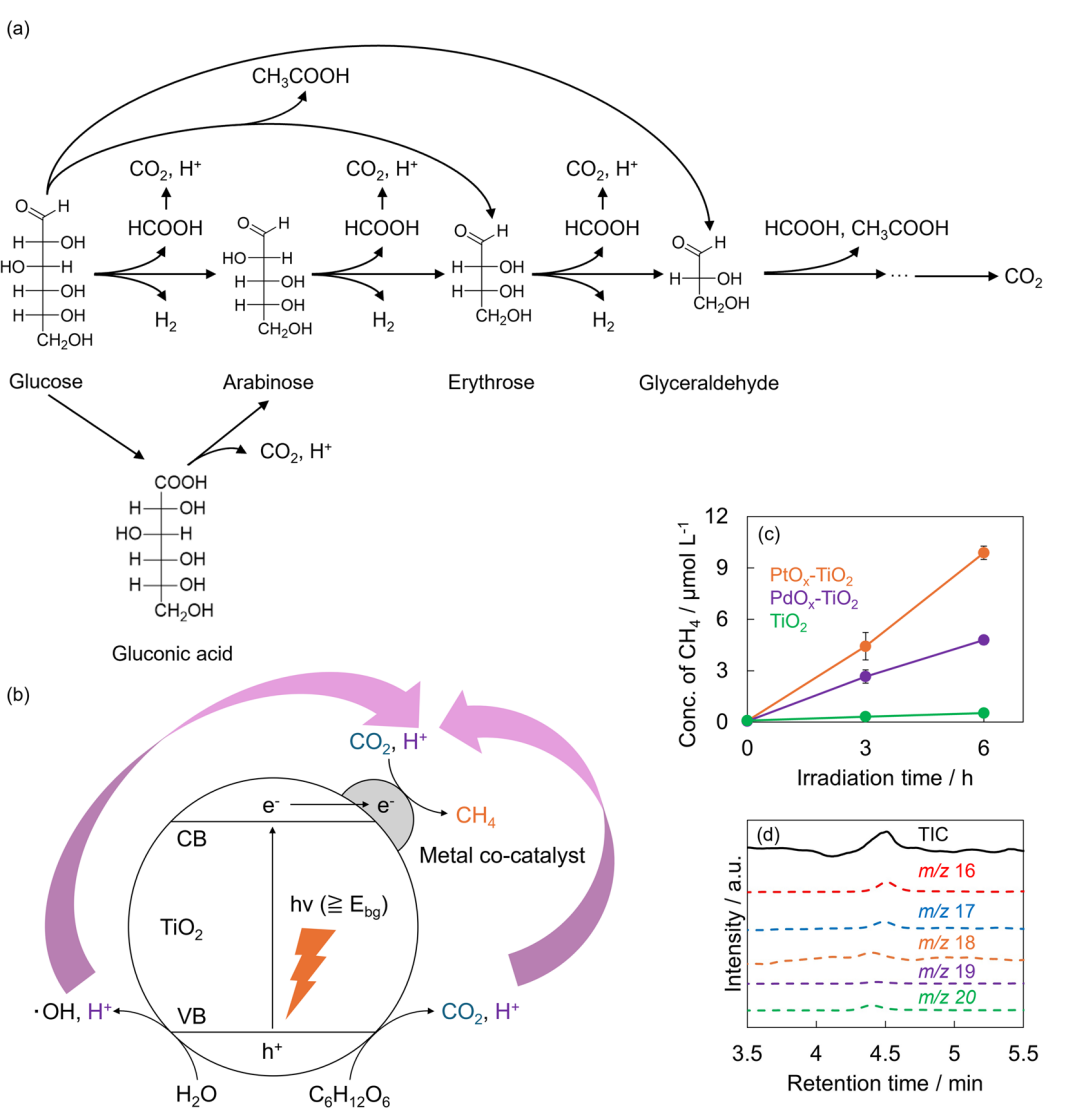
(**a**) Presumable reaction pathways for the photocatalytic decomposition of glucose. (**b**) Schematic illustration of CH_4_ production through glucose decomposition using metal co-catalyst-loaded TiO_2_. (**c**) CH_4_ production using CO_2_ as the starting material (*n* = 3, orange: PtO_x_-TiO_2_, purple: PdO_x_-TiO_2_, green: TiO_2_). (**d**) Mass spectra of CH_4_ generated through photocatalytic degradation of glucose in D_2_O solution (black: TIC, red: *m/z* 16, blue: *m/z* 17, orange: *m/z* 18, purple: *m/z* 19, green: *m/z* 20).

The photocatalytic degradation of glucose produced CO_2_ and H_2_, sugars such as arabinose, erythrose, and glyceraldehyde, and organic acids including formic acid, acetic acid, and gluconic acid. The presumed reaction pathways for glucose degradation based on these results are shown in Fig. 4a. The photocatalytic degradation of glucose proceeds via several distinct reaction pathways. The first reaction pathway involves gluconic acid as an intermediate. Initially, glucose is oxidized to produce gluconic acid, which then undergoes decarboxylation to generate arabinose^75^. During the decarboxylation of gluconic acid, CO_2_ and H^+^ are formed as byproducts^75^. The second reaction pathway involves direct generation of arabinose through C1-C2 bond cleavage of glucose. The third reaction pathway encompasses erythrose formation through C2-C3 bond cleavage of glucose, as well as glyceraldehyde formation through C3-C4 bond cleavage of glucose. The arabinose generated in the first and second pathways proceeds through sequential reactions, wherein formic acid elimination produces erythrose, and further formic acid elimination from erythrose produces glyceraldehyde. During these sequential elimination reactions, H_2_ is formed as a byproduct^75^. Subsequently, the oxidation of glyceraldehyde leads to acetic acid formation, and when complete oxidation of glyceraldehyde occurs, CO_2_ is ultimately generated. Thus, the photocatalytic degradation of glucose produces sugars, organic acids, and H_2_, while simultaneously generating CO_2_ and H^+^.

The preceding experiments elucidated the production pathways of sugars, organic acids, CO_2_ and H^+^ during photocatalytic glucose decomposition; however, the generation mechanisms underlying CH_4_ formation remain insufficiently characterized. Consequently, based on accumulated experimental evidence, we examined the mechanisms governing CH_4_ generation during glucose decomposition using metal co-catalyst-loaded TiO_2_ photocatalysts (Fig. 4b). Upon photoirradiation, the holes generated within the valence band of TiO_2_ reacted with water molecules to form hydroxyl radicals as reactive oxygen species. Concurrently, the photoexcited electrons generated in the conduction band interact with molecular oxygen to produce superoxide anion radicals, which are also reactive oxygen species. These photogenerated holes and reactive oxygen species facilitate glucose oxidation, ultimately yielding CO_2_ and H^+^ through sequential intermediates comprising sugars and organic acids. Furthermore, H^+^ is concomitantly generated along with hydroxyl radicals during water oxidation. The resultant H^+^ ions undergo reduction by photoexcited electrons in the conduction band, thereby forming H_2_. Subsequently, the CO_2_ and H^+^ species produced through these photocatalytic processes are anticipated to undergo methanation reactions at the metal co-catalyst surface, culminating in CH_4_ formation.

To verify whether CH_4_ generation proceeds via the proposed methanation mechanism, we conducted controlled experiments under photoirradiation conditions using CO_2_ as the sole carbon source instead of glucose. The experimental protocol involved the addition of 20 mL of ultrapure water and 20 mg of photocatalyst to a sealed reaction vessel equipped with a septum-fitted lid for gas sampling. Subsequently, CO_2_ was introduced into the solution by bubbling for 30 min to achieve saturation, followed by UV irradiation of the reaction system. Gas-phase samples were collected prior to photoirradiation and at 3 h and 6 h intervals following irradiation initiation. Quantitative CH_4_ analysis was performed using GC. The experimental results are presented in Fig. 4c. CH_4_ generation was observed following photoirradiation initiation, with production quantities after 6 h measuring 9.875 µmol L^− 1^ for PtO_x_-TiO_2_, 4.784 µmol L^− 1^ for PdO_x_-TiO_2_, and 0.525 µmol L^− 1^ for pristine TiO_2_. Confirmation of CH_4_ generation in the absence of added glucose conclusively demonstrates that methanation reactions occur between photocatalytically generated H^+^ and dissolved CO_2_, thereby establishing a mechanistic pathway for CH_4_ production.

A control experiment under dark conditions was conducted to confirm that the observed CH_4_ generation depends on photocatalytic reactions, specifically demonstrating that CH_4_ is not produced in the absence of light irradiation, even when CO_2_ is present in the environment. To maintain the experimental conditions as similar as possible to those under light irradiation, glucose at 25 mmol L^− 1^ was dissolved in 20 mL of ultrapure water, although glucose was not required for this particular reaction. Subsequently, 20 mg of photocatalyst was added to the reaction vessel. The reaction vessel was sealed using a lid equipped with a septum for gas sampling. Pure hydrogen (99.99%) and pure CO_2_ (99.9%) were added to 50 µL of the reaction substrate, and the reaction was conducted under dark conditions without light irradiation. Gas samples (1.0 mL) were collected from the gas phase of the reaction vessel before substrate addition, 3 h after addition, and 6 h after addition, followed by the quantitative analysis of CH_4_ using gas chromatography. As shown in Figure S13, CH_4_ generation was below the detection limit under dark conditions when either PtO_x_-TiO_2_ or PdO_x_-TiO_2_ was used. These results clearly demonstrate that the CH_4_ generation observed in this study was induced by photocatalytic processes.

To identify the sources of hydrogen atoms that constitute CH_4_ molecules with the aim of elucidating the detailed mechanism of CH_4_ generation through photocatalytic reactions, deuterium-labeling experiments were conducted. Specifically, 20 mL of D_2_O containing 25 mmol L^− 1^ glucose and 20 mg of PtO_x_-TiO_2_ were added to a closed reaction vessel and irradiated with UV light (light irradiation intensity: 50 mW cm^− 2^). Gas generated in the reaction system was sampled 6 h after the start of light irradiation, and molecular weight analysis was performed using GC-MS. The analysis results are shown in Fig. 4d. methane corresponding to molecular weights of 16 (CH_4_), 17 (CH_3_D), 18 (CH_2_D_2_), 19 (CHD_3_), and 20 (CD_4_) was detected, with R.T. of 4.52 min, 4.50 min, 4.41 min, 4.44 min, and 4.41 min, respectively. The detection of chemical species in which hydrogen atoms in CH_4_ molecules were substituted with deuterium (CH_3_D, CH_2_D_2_, CHD_3_, and CD_4_) confirmed that the D^+^ involved in methane generation was supplied by D_2_O. Meanwhile, the detection of unlabeled CH_4_ suggests that hydrogen species were also supplied by the glucose molecules themselves. These results reveal that the sources of H^+^ in the photocatalytic glucose decomposition originate from both water and glucose.

In Fig. 2b, PtO_x_-TiO_2_ reached a plateau after 3 h of light irradiation. On the other hand, when CO_2_ solution was used as the starting material, the CH_4_ production on PtO_x_-TiO_2_ (Fig. 4c) increased continuously, rather than plateauing. Specifically, the CH_4_ concentration was approximately 4.357 µmol L^− 1^ during the first 3 h of UV irradiation and further increased to 5.444 µmol L^− 1^ between 3 and 6 h. This behavior may be attributed to differences in the availability of CO_2_, which serves as the carbon source for CH_4_ formation. Figure S14 shows the temporal profiles of CO_2_ concentrations for reactions starting from CO_2_ and from glucose, on PtO_x_-TiO_2_. In the case of glucose decomposition with PtO_x_-TiO_2_, the CO_2_ concentration increased to approximately 599 µmol L^− 1^ during the first 3 h of irradiation but decreased to 350 µmol L^− 1^ between 3 and 6 h. In contrast, for CO_2_ conversion using PtO_x_-TiO_2_, the CO_2_ concentration in the reactor was approximately 21,150 µmol L^− 1^ before irradiation, 19,186 µmol L^− 1^ at 3 h, and 19,403 µmol L^− 1^ at 6 h, indicating that sufficient CO_2_ was available compared with the glucose decomposition case. These results suggest that, in CH_4_ production from glucose decomposition using PtO_x_-TiO_2_, the platoo observed in CH_4_ formation after 3 h of irradiation can be partly attributed to insufficient CO_2_ supply for the methanation. In addition, in this study, it was also possible that the decomposition reaction of the CH_4_ occurred on PtO_x_-TiO_2_ systems^76^, which suggested that the generation and degradation rates of CH_4_ might have become comparable after 3 h of light irradiation.

Table S2 represents a comparison between this work and previous studies regarding CH_4_ yields in photocatalytic degradation of glucose. In CH_4_ production via photocatalytic degradation of glucose, previous studies have shown that Pd loading effectively promotes CH_4_ production^62^. However, this work demonstrated the CH_4_ production with not only PdO_x_ but also that the PtO_x_ loading systems and exhibited that PtO_x_-TiO_2_ showed CH_4_ yields ca. ten times higher than those of the PdO_x_-TiO_2_. Furthermore, this work suggested that methane is ultimately produced via the methanation of CO_2_, produced via decomposition of glucose, and H^+^, derived from both glucose and water, which was not clarified in previous studies.

The significant difference in CH_4_ generation between PtO_x_-TiO_2_ and PdO_x_-TiO_2_ observed in this study originates from the fundamental differences in the electronic and chemical properties of the Pt and Pd species. This difference in performance can be explained by two factors. The first is the utilization efficiency of electrons, which serves as the driving force for photocatalytic reactions. PL measurements were conducted to investigate the suppression ability of carrier recombination in the prepared photocatalysts. Figure S15 shows the PL spectra of PtO_x_-TiO_2_, PdO_x_-TiO_2_, and bare TiO_2_. It was found that the PL intensity was the highest for bare TiO_2_, followed by PtO_x_-TiO_2_, and the lowest for PdO_x_-TiO_2_. Electrochemical impedance spectroscopy (EIS) was conducted to further investigate in detail the suppression of carrier recombination by the supported metals. Figure S16 shows the Nyquist plots of PtO_x_-TiO_2_, PdO_x_-TiO_2_, and bare TiO_2_ under light irradiation, obtained from electrochemical impedance measurements. All photocatalysts exhibited Nyquist plots with a compressed semicircular feature within the measured range, corresponding to high- and mid-frequency domains that reflect charge-transfer processes at the photocatalyst–electrolyte interface. The semicircle radii decreased in the order of TiO_2_ > PdO_x_-TiO_2_ > PtO_x_-TiO_2_. The equivalent circuit used for fitting is shown in the upper-left corner of Figure S16. Rₛ_r_ represents the solution resistance, corresponding to the distance between the origin and the first point in the plot. The constant phase element (CPE) represents a pseudo-capacitance associated with the curvature of the semicircle, while Rₜ_r_ denotes the charge-transfer resistance between the photocatalyst and the electrolyte, which corresponds to the semicircle radius^77^. Among the samples, PtO_x_-TiO_2_ exhibited the smallest semicircle radius, indicating the lowest charge-transfer resistance and hence the most efficient interfacial charge transfer, followed by PdO_x_-TiO_2_ and bare TiO_2_. These results confirm that PtO_x_ loading effectively suppresses electron-hole recombination by promoting charge separation and interfacial transport. Since Pt possesses a larger work function than Pd^78^, it functions as a more powerful electron trap site at the interface with TiO_2_ and efficiently captures photoexcited electrons. This superior charge separation efficiency provided abundant electrons available for reduction reactions on the Pt co-catalyst surface, thereby improving the overall efficiency of the methanation reaction. The second factor is the reaction selectivity of the co-catalyst surface. CH_4_ generation requires the cleavage of stable C-O bonds in CO_2_, which is a reaction with a high-energy barrier^60^. According to d-band center theory, Pt has a higher d-band center than Pd and can more strongly adsorb and activate CO_2_ and its reaction intermediates^79^. This strong interaction promotes the cleavage of the carbon-oxygen bonds, which is the rate-determining step of methanation. In contrast, the relatively weak adsorption capability of Pd makes carbon-oxygen bond cleavage difficult, causing electrons to be preferentially consumed in the competing hydrogen evolution reaction, which proceeds more readily. This is consistent with the experimental results showing that the PdO_x_-TiO_2_ system generated more H_2_ than the PtO_x_-TiO_2_ system. In conclusion, the synergistic effect of the superior electron-capture capability of Pt and its inherently high selectivity toward methanation reactions creates an advantage in CH_4_ generation over Pd.

## Conclusions

This study successfully demonstrated sustainable CH_4_ production through photocatalytic glucose degradation under ambient conditions. Mechanistic investigations revealed that photocatalytic glucose degradation proceeds via complex reaction pathways involving intermediate sugars and organic acids. These compounds undergo further oxidation to generate CO_2_ and H^+^. CH_4_ formation occurs primarily through the methanation of internally produced CO_2_ and H^+^, which are reduced on the metal co-catalyst surface by photoexcited electrons. This mechanism was validated through control experiments, including CH_4_ production from CO_2_-saturated solutions under irradiation, and confirming that no CH_4_ generation occurs under dark conditions. The theoretical potential of glucose as a carbon source, which yields up to six CH_4_ molecules per glucose molecule, highlights the efficiency of this photocatalytic approach. Although current conversion rates require optimization for practical implementation, this research establishes the fundamental feasibility of photocatalytic biomass-to-fuel conversion under mild conditions.

## Supplementary Information

Below is the link to the electronic supplementary material.

Supplementary Material 1

## Data Availability

All data generated or analyzed during this study are included in this published article.
