# Supplementary material for: Methane production via photocatalytic degradation of glucose on PtOx and PdOx-loaded TiO2
Source: Sci Rep. 2025 Dec 3;16:721. doi: 10.1038/s41598-025-30321-w (PMC12780279; doi:10.1038/s41598-025-30321-w)
Supplement: Supplementary file 1 — Supplementary Material 1 [file 41598_2025_30321_MOESM1_ESM.docx]

**Supplementary information**

**Methane production via photocatalytic degradation of glucose on
PtO_x_ and PdO_x_-loaded TiO_2_**

Yuma Uesaka^1^, Kio Kawakatsu^1^, Mana Akita^1^, Toshiya Tsunakawa^1^, Satoki Yoshida^1^, Naoko Taki^1^, Tiangao Jiang^1^, Shanhu Liu^2^, Eika W. Qian^1^, Sho Usuki^1,*^, Kazuya Nakata^1,*^

^1^Graduate School of Bio-Applications and Systems Engineering, Tokyo University of Agriculture and Technology, 2-24-16 Naka-cho, Koganei, Tokyo 184-0012, Japan

^2^Henan Joint International Research Laboratory of Environmental Pollution Control Materials, Henan Key Laboratory of Polyoxometalate Chemistry, College of Chemistry and Chemical Engineering, Henan University, Kaifeng, 475004, PR China

**
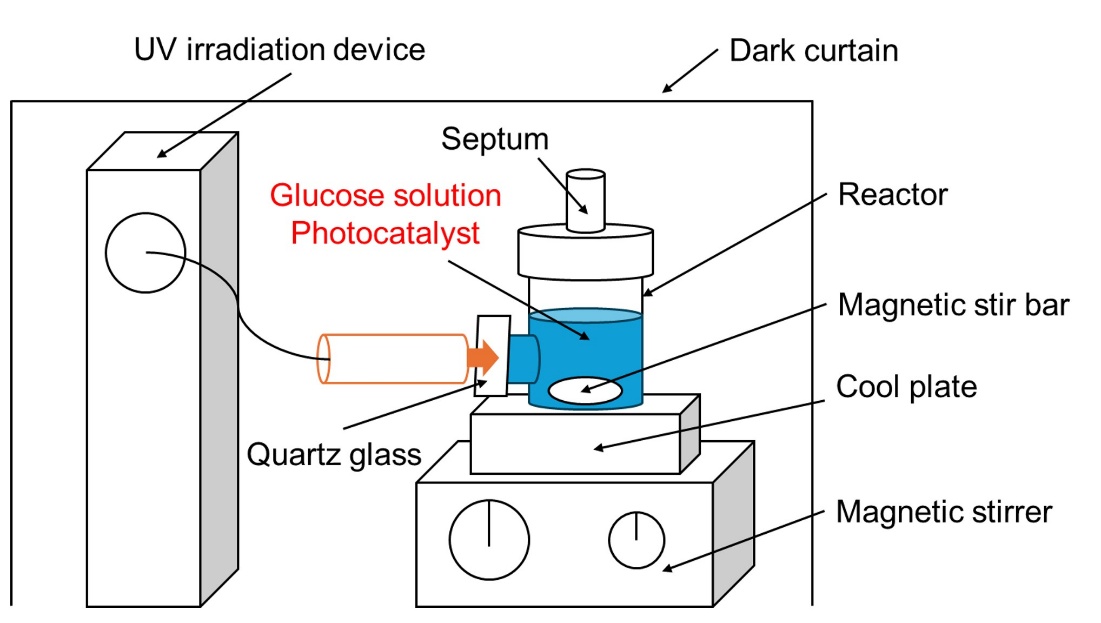
**

**Figure S1.** Schematic overview of the experimental setup.


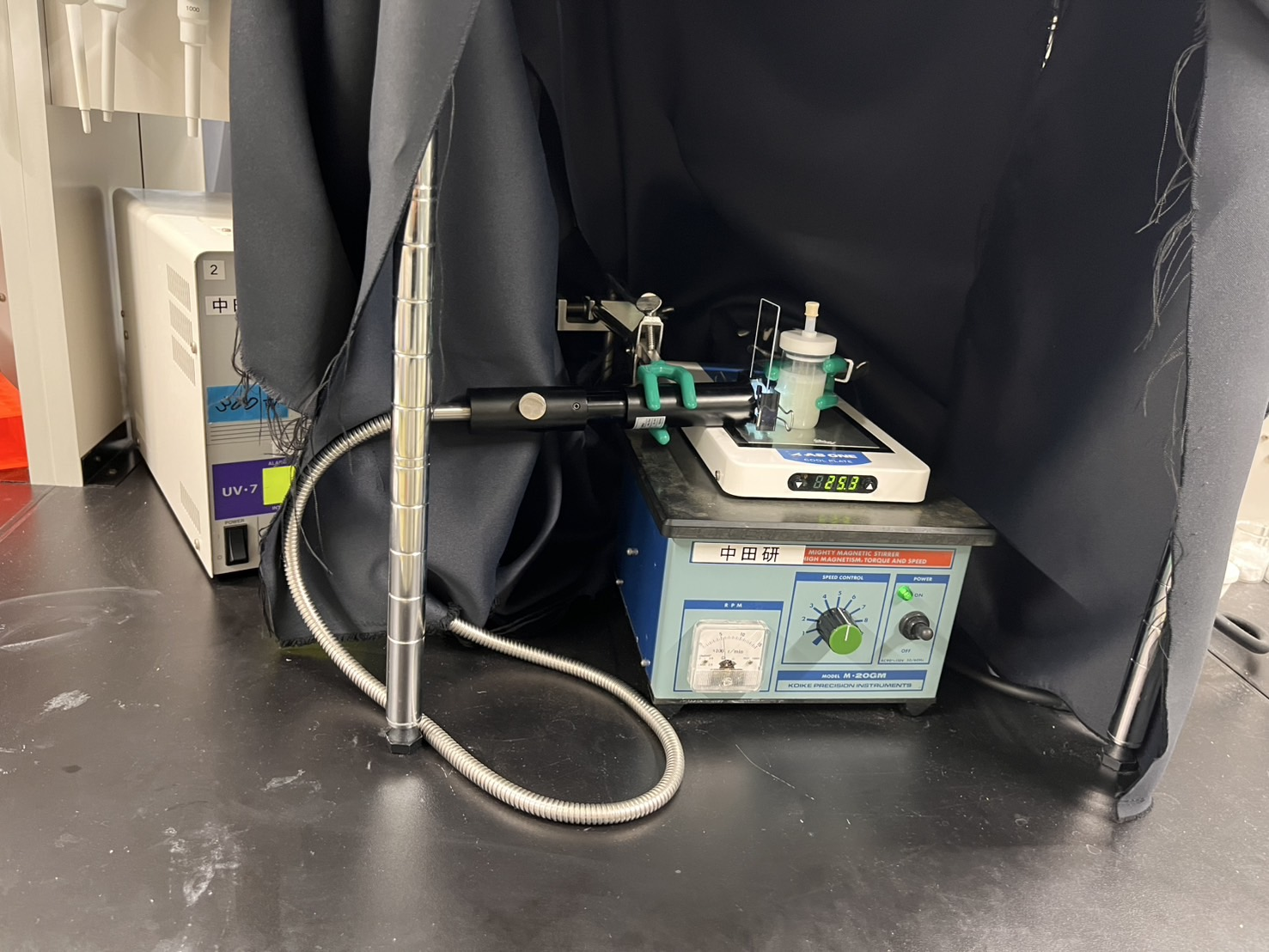


**Figure S2.** Picture of the experimental setup.

**
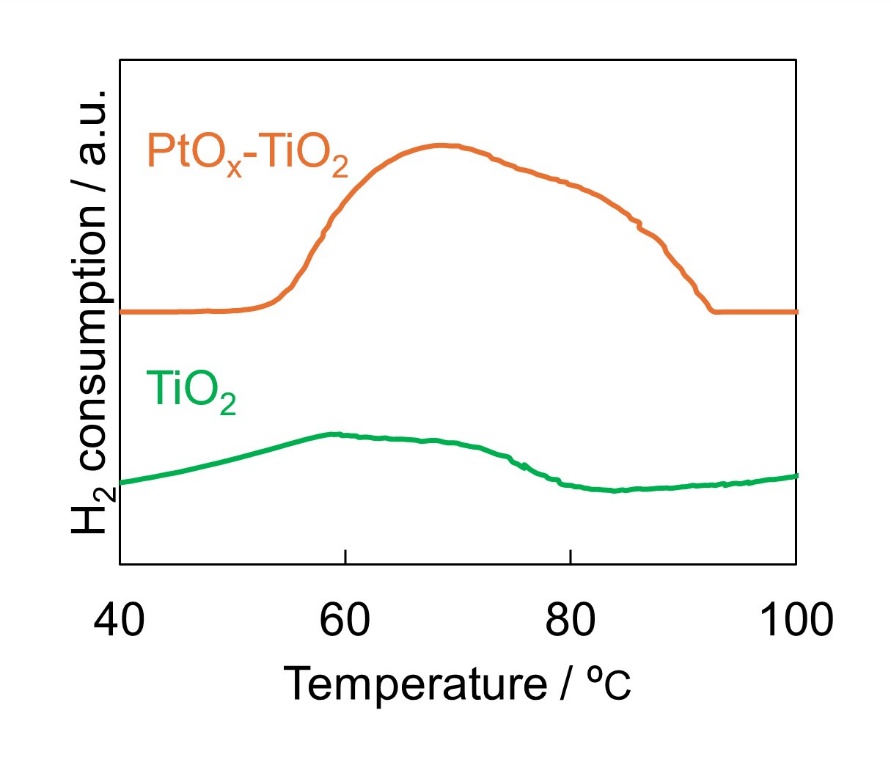
**

**Figure S3.** H_2_-TPR profiles (orange: PtO_x_-TiO_2_, green: TiO_2_).

**
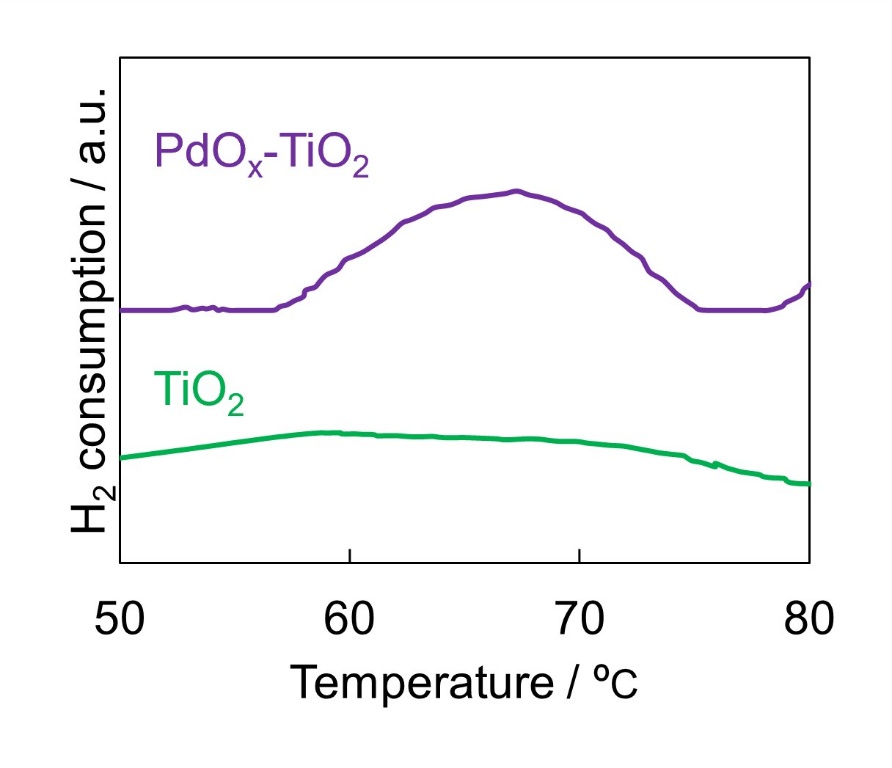
**

**Figure S4.** H_2_-TPR profiles (purple: PdO_x_-TiO_2_, green: TiO_2_).

**
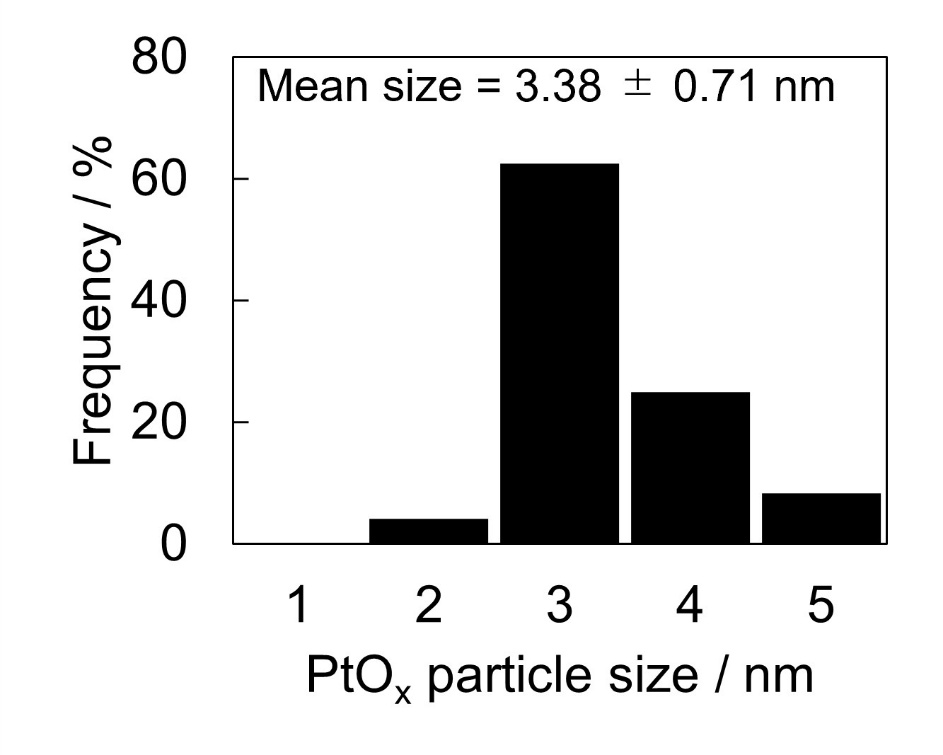
**

**Figure S5.** PtO_x_ particle size distributions for PtO_x_-TiO_2_.

**
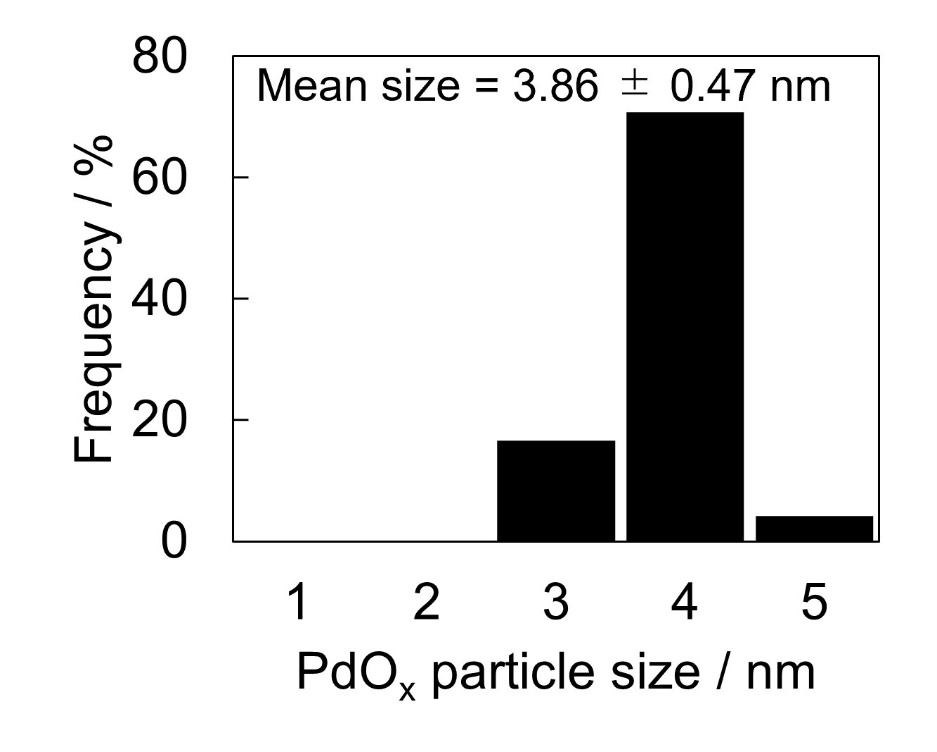
**

**Figure S6.** PdO_x_ particle size distributions for PdO_x_-TiO_2_.

**
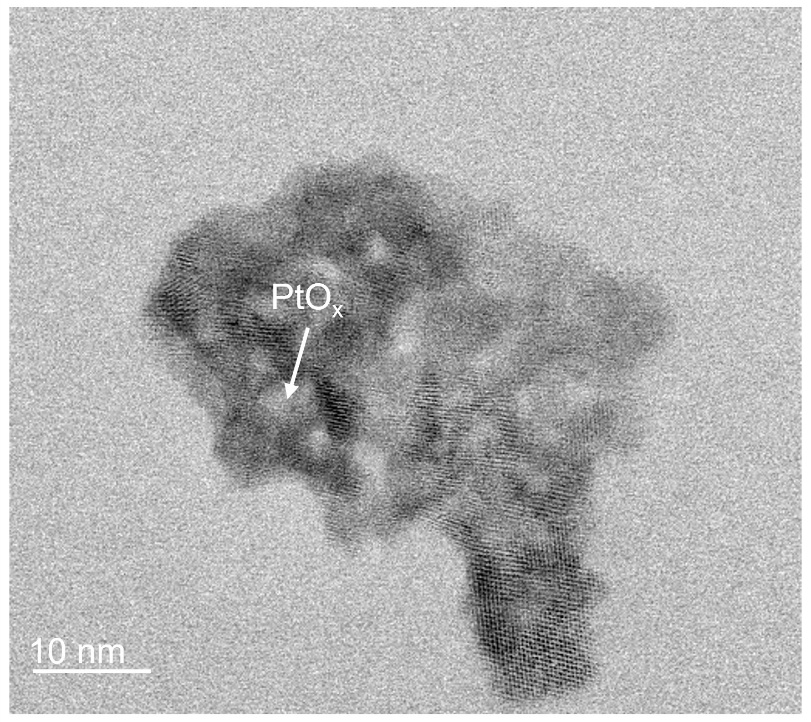
**

**Figure S7.** STEM image of 0.5 wt% PtO_x_-TiO_2_ before light irradiation.

**
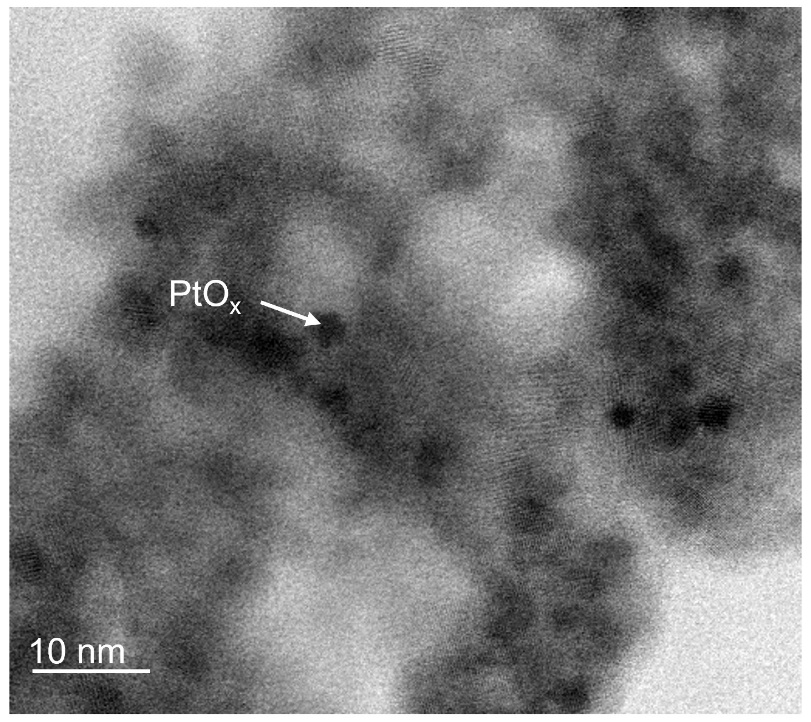
**

**Figure S8.** STEM image of 0.5 wt% PtO_x_-TiO_2_ after light irradiation.

**
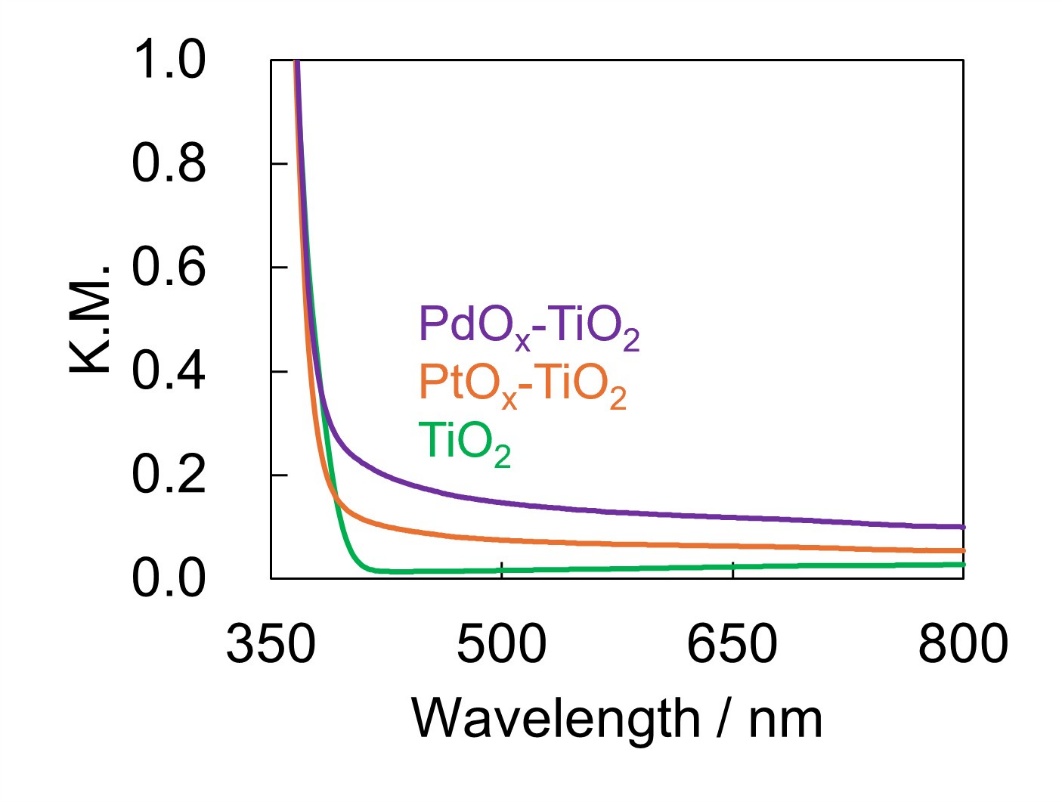
**

**Figure S9.** UV-vis spectra of photocatalysts (orange: PtO_x_-TiO_2_, purple: PdO_x_-TiO_2_, green: TiO_2_).

**
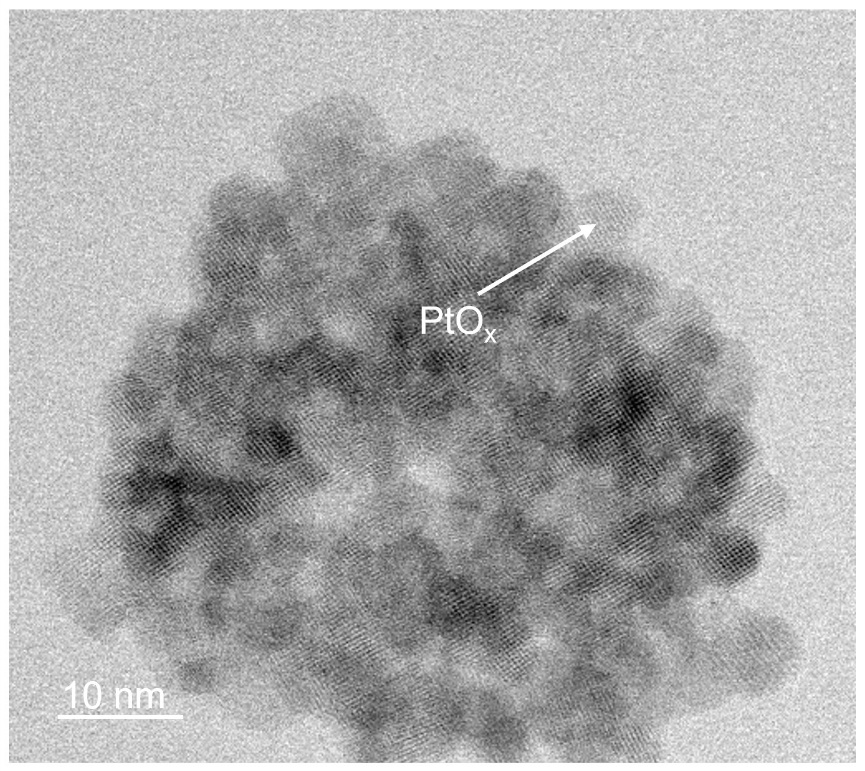
**

**Figure S10.** STEM image of 2.0 wt% PtO_x_-TiO_2_.

**
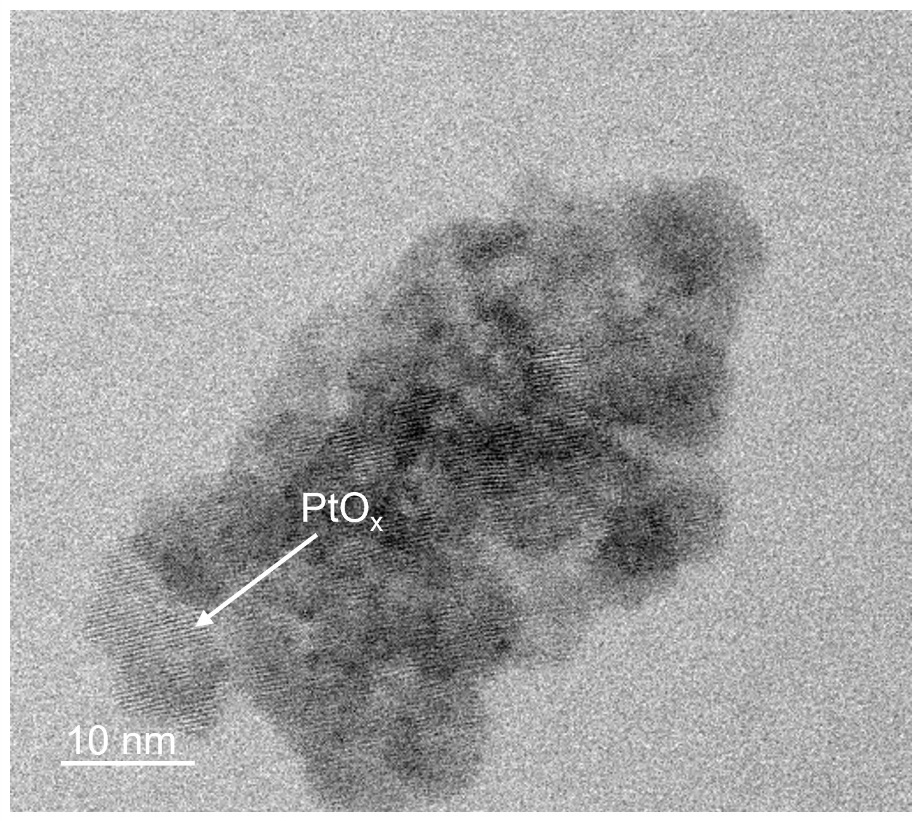
**

**Figure S11.** STEM image of 4.0 wt% PtO_x_-TiO_2_.

**
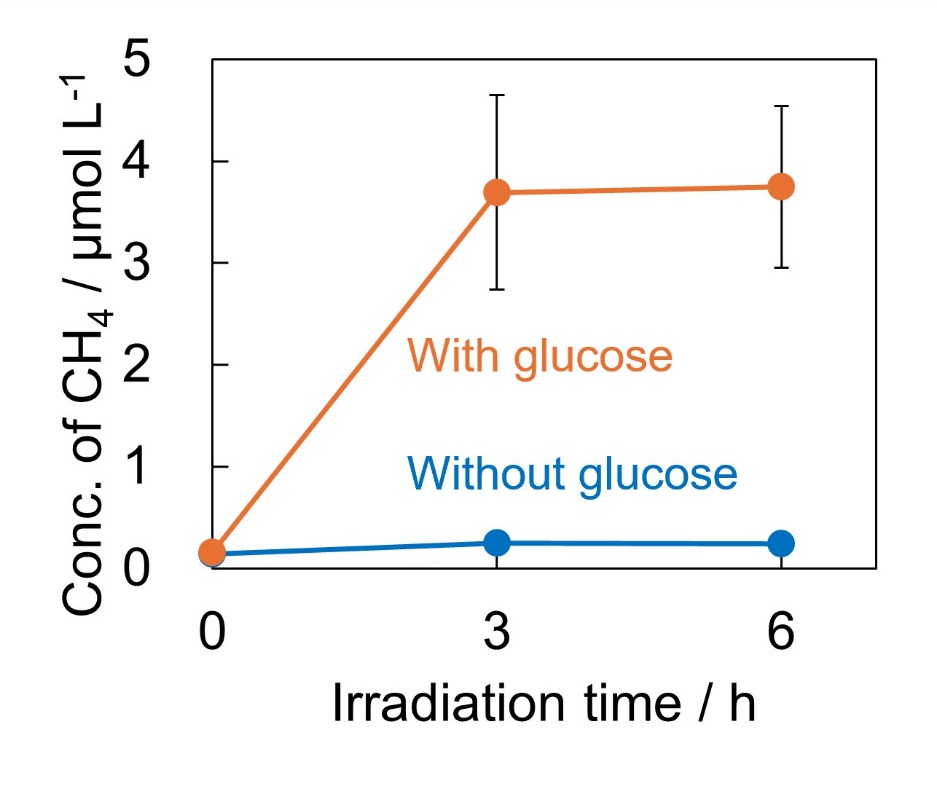
**

**Figure S12.** Time course of CH_4_ concentrations (n=3, orange: with glucose, blue: without

glucose).


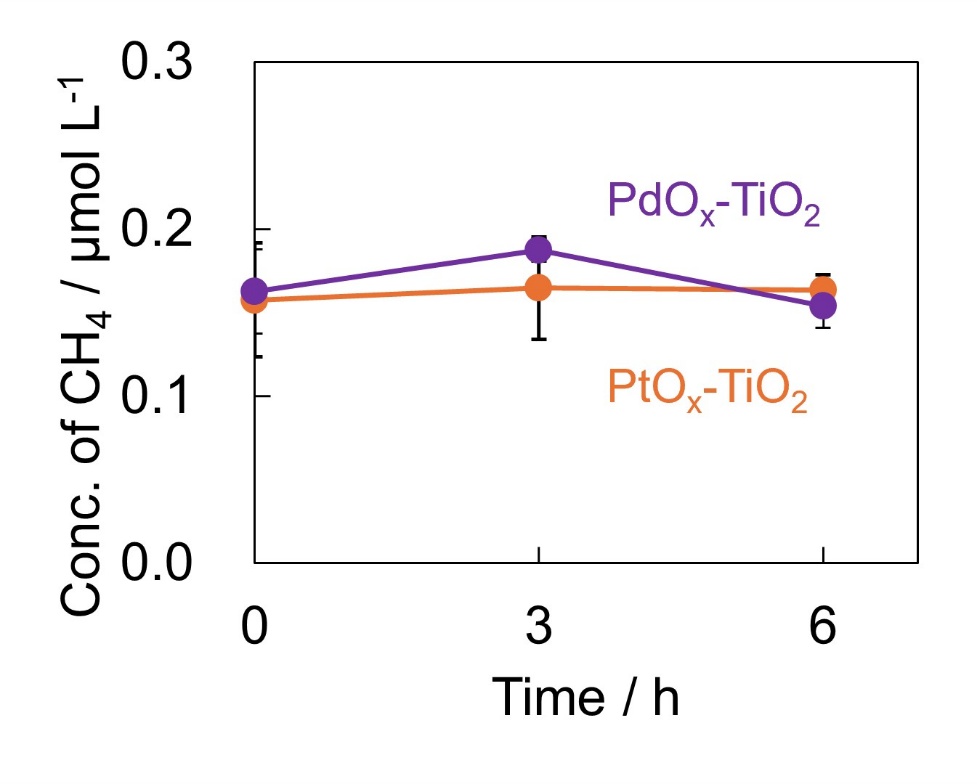


**Figure S13.** Time course of CH_4_ concentrations in catalytic reaction experiments using

CO_2_ and hydrogen with PtO_x_-TiO_2_ (orange) and PdO_x_-TiO_2_ (purple) in the dark (n=3).


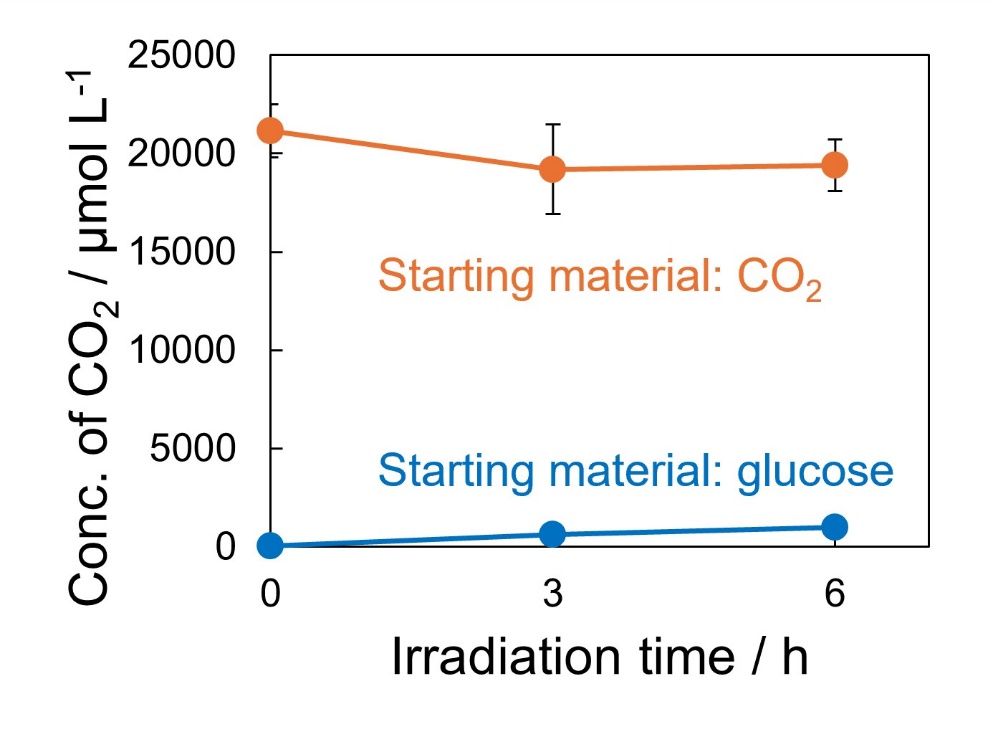


**Figure S14.** Time course of CO_2_ concentration (starting material: CO_2_ (orange), glucose (blue)).


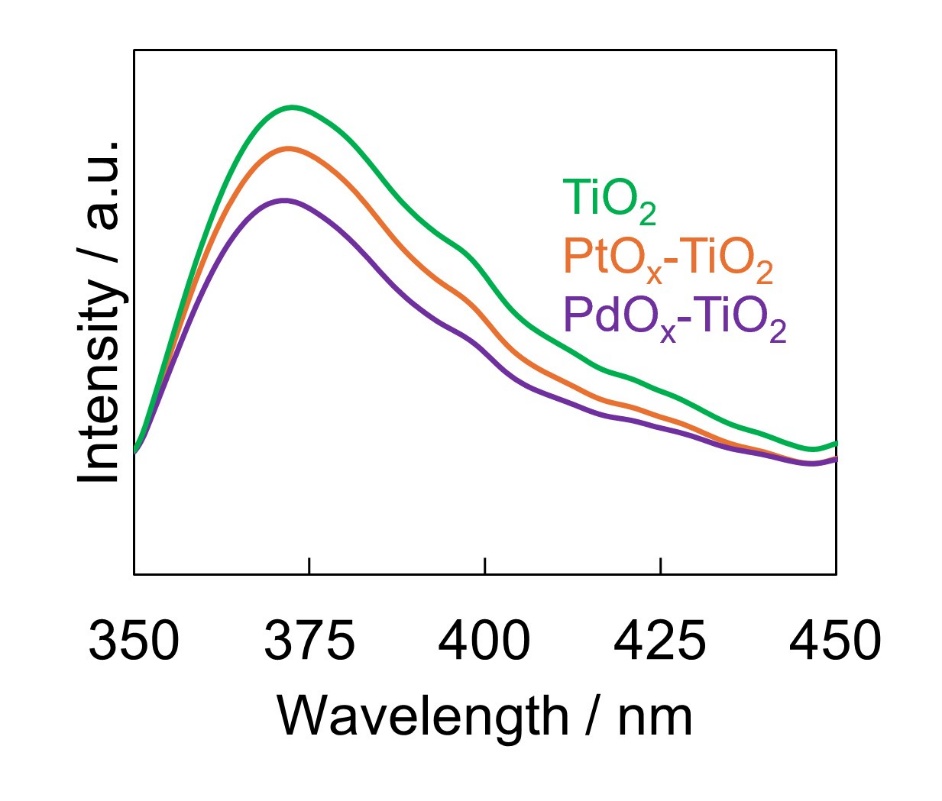


**Figure S15.** PL spectra of photocatalysts (orange: PtO_x_-TiO_2_, purple: PdO_x_-TiO_2_, green: TiO_2_).


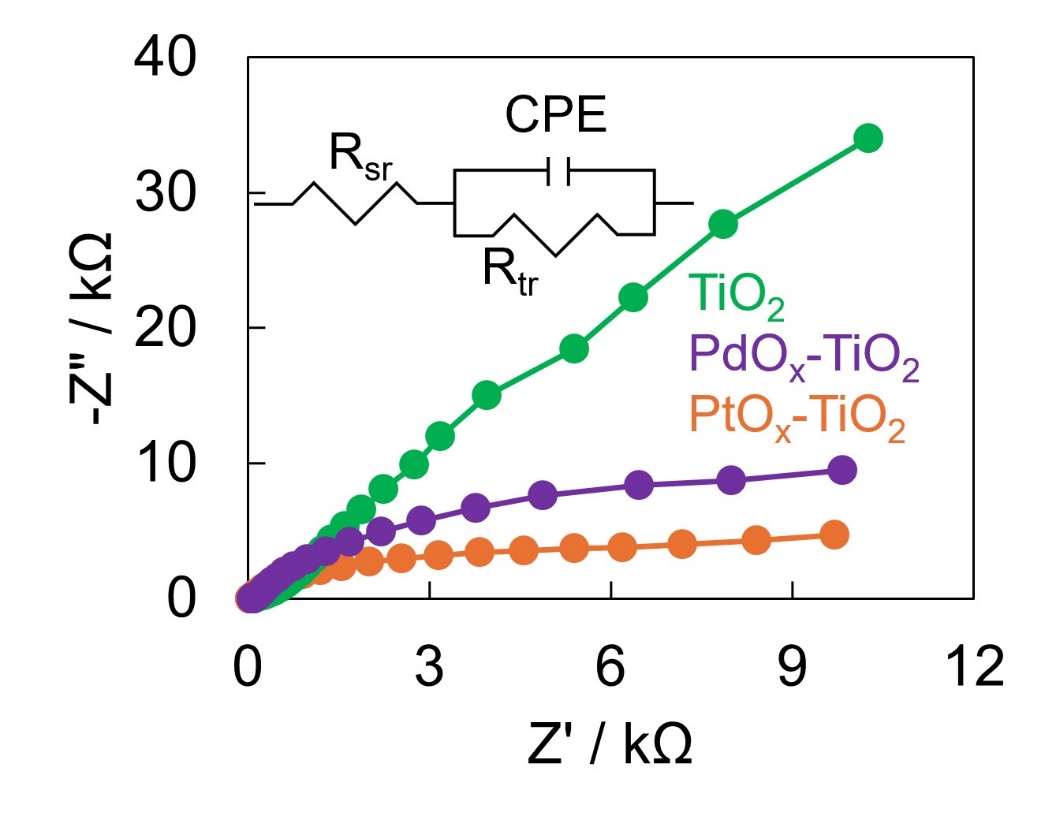


**Figure S16.** EIS Nyquist plots (orange: PtO_x_-TiO_2_, purple: PdO_x_-TiO_2_, green: TiO_2_).

**Table S1.** Carbon balance after 6 h of light irradiation (Unit: %, n=3).

|  | PtO_x_-TiO_2_ | PdO_x_-TiO_2_ | TiO_2_ | No photocatalyst |
| --- | --- | --- | --- | --- |
| Glucose | 86.64 | 92.17 | 92.30 | 99.56 |
| CO_2_ | 0.67 | 0.44 | 0.25 | 0.027 |
| CH_4_ | 0.0025 | 0.00014 | - | - |
| Arabinose | 4.93 | 3.49 | 1.89 | 0.055 |
| Erythrose | 0.33 | 0.27 | 0.012 | - |
| Glyceraldehyde | 0.22 | 0.16 | 0.022 | - |
| Formic acid | 1.89 | 2.00 | 0.047 | 0.015 |
| Total | 94.69 | 98.52 | 95.18 | 99.69 |

**Table S2.** Comparison between previous studies and this work regarding CH_4_ yields in photocatalytic degradation of glucose.

|  | This work | Vaiano (2015)^62^ |
| --- | --- | --- |
| Solution | 4600 mg L^-1^ Glucose 20 mL | 500 mg L^-1^ Glucose 80 mL |
| Light Intensity | 50 mW cm^-2^ | 0.15 mW cm^-2^ |
| Photocatalyst | 2.0 wt%PtO_x_-TiO_2_ | 0.5 wt%Pd-TiO_2_ |
| Photocatalyst dosage | 20 mg | 40 mg |
| CH_4_ yield after 3h of irradiation | 0.04 µmol | 7.58 µmol |
